# Evolutionary History of Copper Membrane Monooxygenases

**DOI:** 10.3389/fmicb.2018.02493

**Published:** 2018-10-29

**Authors:** Roshan Khadka, Lindsay Clothier, Lin Wang, Chee Kent Lim, Martin G. Klotz, Peter F. Dunfield

**Affiliations:** ^1^Department of Biological Sciences, University of Calgary, Calgary, AB, Canada; ^2^Department of Biological Sciences, University of North Carolina at Charlotte, Charlotte, NC, United States; ^3^School of Molecular Biosciences, College of Veterinary Medicine, Washington State University, Richland, WA, United States; ^4^State Key Laboratory of Marine Environmental Science, Institute of Marine Microbes and Ecospheres, College of Ocean and Earth Sciences, Xiamen University, Xiamen, China

**Keywords:** methane oxidation, ammonia oxidation, copper membrane monooxygenase, lateral gene transfer, phylogenetics

## Abstract

Copper membrane monooxygenases (CuMMOs) oxidize ammonia, methane and some short-chain alkanes and alkenes. They are encoded by three genes, usually in an operon of *xmoCAB*. We aligned *xmo* operons from 66 microbial genomes, including members of the *Alpha*-, *Beta*-, and *Gamma-proteobacteria, Verrucomicrobia, Actinobacteria, Thaumarchaeota* and the candidate phylum NC10. Phylogenetic and compositional analyses were used to reconstruct the evolutionary history of the enzyme and detect potential lateral gene transfer (LGT) events. The phylogenetic analyses showed at least 10 clusters corresponding to a combination of substrate specificity and bacterial taxonomy, but with no overriding structure based on either function or taxonomy alone. Adaptation of the enzyme to preferentially oxidize either ammonia or methane has occurred more than once. Individual phylogenies of all three genes, *xmoA, xmoB* and *xmoC*, closely matched, indicating that this operon evolved or was consistently transferred as a unit, with the possible exception of the methane monooxygenase operons in *Verrucomicrobia*, where the *pmoB* gene has a distinct phylogeny from *pmoA* and *pmoC*. Compositional analyses indicated that some clusters of *xmoCAB* operons (for example, the *pmoCAB* in gammaproteobacterial methanotrophs and the *amoCAB* in betaproteobacterial nitrifiers) were compositionally very different from their genomes, possibly indicating recent lateral transfer of these operons. The combined phylogenetic and compositional analyses support the hypothesis that an ancestor of the nitrifying bacterium *Nitrosococcus* was the donor of methane monooxygenase (pMMO) to both the alphaproteobacterial and gammaproteobacterial methanotrophs, but that before this event the gammaproteobacterial methanotrophs originally possessed another CuMMO (Pxm), which has since been lost in many species.

## Introduction

The copper membrane monooxygenase (CuMMO) enzyme family includes ammonia monooxygenase (AMO), particulate methane monooxygenase (pMMO) and a few short-chain alkane and alkene monooxygenases (Tavormina et al., 2011). AMO and pMMO perform the initial oxygenation steps in the aerobic catabolism of methane (CH_4_) and ammonia (NH_3_), which produce toxic intermediates (methanol, formaldehyde, and/or hydroxylamine) that constitute the internal sources of energy and reductant for these microbes (Klotz and Stein, 2008, 2011; Simon and Klotz, 2013). Organisms with these enzymes therefore play key roles in the global carbon and nitrogen cycles. Substrates other than methane and ammonia are also targeted by some CuMMOs: some *Actinobacteria* possesses a putative CuMMO that acts as a butane monooxygenase (BMO) (Coleman et al., 2011, 2012; Sayavedra-Soto et al., 2011), and ethylene-assimilating *Gammaproteobacteria* belonging to the genus *Haliea* also possess CuMMOs (Suzuki et al., 2012). Genomics projects are also beginning to find operons encoding CuMMOs of unknown function in bacterial genomes, such as *Solimonas aquatica* and *Bradyrhizobium* sp. ERR11 (Kyrpides et al., 2014; Whitman et al., 2015). The known diversity of the CuMMO enzyme family is therefore expanding. CuMMOs are promiscuous and co-oxidize several structurally similar substrates, although they usually show clear specialization to one particular substrate (Bedard and Knowles, 1989; Semrau et al., 2010). For example, pMMO will oxidize ammonia and some alkanes, but usually with a lower affinity and lower reaction rates than for methane (Bedard and Knowles, 1989; Nyerges and Stein, 2009). This co-oxidation of ammonia and the resulting production of toxic hydroxylamine and nitrite leads to an inhibitory effect of ammonia on methanotrophs (Bodelier and Laanbroek, 2004; Nyerges and Stein, 2009).

Copper membrane monooxygenase enzymes are encoded by three genes (Klotz et al., 1997), here referred to collectively as *xmoC, xmoA*, and *xmoB* (*amo* specifically refers to genes encoding AMO, and *pmo* specifically refers to genes encoding pMMO). A clear homology of *amoAB*/*pmoAB* genes was first shown by Klotz and Norton (1998), demonstrating that AMO and pMMO were evolutionarily related. In bacteria, the *xmoAB* genes are always clustered with an *xmoC*, although *xmoC* genes sometimes occur as singletons in addition to operonal copies (Klotz et al., 1997; Arp et al., 2007; Op den Camp et al., 2009; Dam et al., 2012). In ammonia oxidizing bacteria, AMO is encoded by an *amoCAB* operon, but in *Thaumarchaeota* the *amoA, amoB*, and *amoC* gene are not always clustered together. A pMMO is encoded in the genomes of nearly all known aerobic methane oxidizing bacteria as a *pmoCAB* operon. Another ortholog present in some proteobacterial methanotrophs has been dubbed *pxmABC*, and the encoding operon is uniquely organized in the ABC order, instead of the more common CAB (Tavormina et al., 2011).

Phylogenies of *amoA*/*pmoA* match closely to 16S rRNA gene phylogenies, at least to the extent that individual genera, families, classes, and phyla can be clearly identified via comparative sequence analysis (Knief, 2015; Alves et al., 2018). Recovery of *pmoA* and *amoA* genes from natural samples has therefore been used extensively to identify species of methanotrophs and ammonia oxidizers in diverse environments (Rotthauwe et al., 1997; McDonald et al., 2008; Tavormina et al., 2013; Dumont, 2014; Knief, 2015). The first published primer set developed to target *pmoA*, A189f/A682r (Holmes et al., 1995), is still widely used as a broad-spectrum primer set for detecting known methanotrophs and discovering new ones (Knief et al., 2003). However, it has become clear via cultivation and genomics studies that these supposed universal primers do not target all *xmoA* genes in nature, for example universal *pmoA* primers do not amplify genes from verrucomicrobial methanotrophs (Op den Camp et al., 2009).

Aerobic methanotrophy is a rare trait, limited to a few monophyletic clusters of bacteria within the *Alphaproteobacteria, Gammaproteobacteria, Verrucomicrobia* and candidate phylum NC10 (Ettwig et al., 2009; Op den Camp et al., 2009). Dissimilatory ammonia oxidation is similarly rare, known in only two clusters of bacteria within the classes *Betaproteobacteria* and *Gammaproteobacteria*, some *Nitrospira*, and some *Thaumarcheota* (Kuypers et al., 2018). This mosaic nature of ammonia and methane-oxidizing taxa, which are constrained to a few monophyletic clusters (based on 16S rRNA gene phylogeny) scattered across several different phyla, indicates that there have been only a few key lateral gene transfer (LGT) events of *xmo* genes. For example, Tamas et al. (2014) suggested that all *Alphaproteobacteria* methanotrophs arose from a single common methylotrophic ancestor that had obtained *pmoCAB* via a single LGT event from a gammaproteobacterium. *Gammaproteobacteria* and *Verrucomicrobia* methanotrophs also appear to be monophyletic based on 16S rRNA genes.

However, some *Gammaproteobacteria* (Tavormina et al., 2011), *Alphaproteobacteria* (Tchawa Yimga et al., 2003; Wartiainen et al., 2006; Vorobev et al., 2014) and *Verrucomicrobia* (Dunfield et al., 2007; Op den Camp et al., 2009) methanotrophs have multiple divergent copies of the *pmo* operon, indicating a more complex history of gene duplication, evolution, and LGT. Two *pmoCAB* operons in *Methylocystis* sp. strain SC2 (59–66% amino acid sequence identity) are suspected to have different methane affinities (Dunfield et al., 2002; Baani and Liesack, 2008). *Methylacidiphilum* spp. have three phylogenetic distinct *pmoCAB* operons as little as 50% amino acid identity to each other, and these show different expression patterns (Op den Camp et al., 2009; Erikstad et al., 2012; Khadem et al., 2012), although the functional differences among the encoded enzymes are not yet known with certainty. A *pxmABC* operon is also found in addition to *pmoCAB* in some proteobacterial methanotrophs. Its function is not clear yet, but transcript levels of *pxmA* increased in *Methylomicrobium album* strain BG8 (Kits et al., 2015a) and *Methylomonas denitrificans* strain FJG1 (Kits et al., 2015b) under nitrite rich and oxygen limited conditions. In addition to utilizing CuMMOs with different substrate affinities and preferences, some *Gammaproteobacteria* and *Betaproteobacteria* with multiple nearly identical copies of *xmoCAB* operons employ differential regulation to preferentially express individual copies upon environmental cues (Stolyar et al., 1999, 2001; Berube et al., 2007; Berube and Stahl, 2012).

Although phylogenetic constructions using *xmoA* are extensively used to delineate species (Knief, 2015; Alves et al., 2018), the evolutionary history of the genes has not been well-elucidated. In this study we assembled a database of *xmoCAB* operons from microbial genomes, and performed in depth compositional and phylogenetic analyses of the genes and their genomes to better understand their evolutionary history.

## Materials and Methods

### Gene and Genome Sequences

All genes and genomes analyzed in this study were downloaded from the JGI^1^, NCBI^2^, or RAST^3^ genomic databases. The database contained 66 genomes, including 4 *Thaumarchaeota*, 50 *Alpha-, Beta-*, and *Gamma-proteobacteria*, and 13 non-proteobacterial genomes (*Verrucomicrobia, Nitrospira*, NC10, and *Actinobacteria*), all of which contained CuMMO-encoding operons. These genomes were chosen based on published papers and BLAST sequences of JGI/NCBI databases. Our aim was to include only cultured bacteria for which physiological data and complete genomes are available. The analyses therefore did not include metagenomic sequences. Some bacterial genomes containing *xmo* genes were also ignored if they appeared to be the result of private sequencing projects with no published reports to date.

### Phylogenetic Constructions

We constructed Bayesian, Maximum likelihood (ML) and Neighbor-Joining (NJ) phylogenies based on derived XmoA, XmoB, XmoC and concatenated XmoCAB protein sequences. The alignment of derived amino acids was done using ClustalW. Seaview version 4.4.12 (Gouy et al., 2010) was used for NJ (with a Poisson evolutionary distance model) and ML (with a Le and Gascuel model) constructions. Bayesian inference methods were calculated with BEAST (Bouckaert et al., 2014), both with strict clock and relaxed clock log normal models. Bayesian analysis employed a Blosum62 substitution model and Gamma site heterogeneity model under a strict clock, or a Gamma site heterogeneity model with four gamma categories with a relaxed clock log-normal model. The inferred tree topology was assessed with 100 bootstrap replications for NJ and ML methods, and 10,000,000 iterations for Bayesian phylogenies minus a burn-in of 20% of the total.

### Compositional Evidence of Lateral Gene Transfer (LGT)

Four programs were used to detect possible LGT of *xmo* operons based on compositional genome biases: TETRA, Alien Hunter, CodonW, and Island Viewer.

FASTA files containing DNA sequences were uploaded into the TETRA program (Teeling et al., 2004b)^4^. Each *xmoCAB* operon and the genome from the respective organism was uploaded separately. Before running the program, the sequences were extended by their reverse complement, and tetra-nucleotide usage patterns were calculated by the program. The program generated a table consisting of all possible 256 tetra-nucleotide combinations with their frequencies in each gene or genome.

The CodonW program (Angellotti et al., 2007), requires coding sequences for calculation. Therefore, the FeatureExtract 1.2 server^5^ was used to extract the coding sequences of a genome from GenBank (.gbk) files. Then the protein coding genes of each *xmoCAB* operon and a genome from each organism were uploaded simultaneously into the CodonW database^6^. The sequences of all coding genes (operons and genomes, separately) were concatenated. The output result contained all the 64 codon bias values. The frequency of each codon was calculated as ‘(codon value)/64’ for each gene or genome separately.

Genomes were uploaded into the Alien Hunter program (Vernikos and Parkhill, 2006) using Linux. The predictions were visualized using Artemis. The program predicts LGT in a moving 2500-bp window, using the Kullback-Leibler (KL) divergence statistic (Kullback and Leibler, 1951) for combined 2-mers to 8-mers.

$$D_{\text{KL}}(region||genome)=\sum_{\text{i}}\ln(\frac{region(i)}{genome(i)})\;region(i)$$

This equation gives numeric values between 0 and 1 as output. Values close to one indicate that the region has a much different compositional bias compared to the genome, and likely underwent horizontal transfer into its genome (Overbeek et al., 2005; Becq et al., 2010). Values close to zero indicate the region has similar kmer composition to its genome (no detectable LGT). Alien Hunter selects a significance KL threshold to determine whether the composition of the operon differs from the genome. The loci of the *xmoCAB* operon(s) in the genomes were used to determine whether these were located within predicted “alien” regions.

For TETRA and CodonW, the KL statistic was also calculated comparing the *xmoCAB* operon(s) to the genome. For TETRA and CodonW we simply report and compare KL values of the *xmoCAB* operon (on average 2730 nucleotides) compared to the composition of the entire genome, without a presumed significance threshold. Individual genes were not tested as they contained insufficient information to accurately predict k-mer frequencies.

Genomes were also examined via Island Viewer (Bertelli et al., 2017), which combines compositional analyses with examination of mobility genes to identify genomic islands.

### Specificity of Different Primer Sets

Several primers that are specific to detect methanotrophs have been developed (Dumont, 2014; Wang et al., 2017). We have analyzed the sensitivity of primers to different groups of methanotrophs (Supplementary Table S2) that are used in this study. We used the Arb platform to detect the number of mismatches in the existing primer sets (Ludwig et al., 2004).

## Results

### Phylogenetic Analyses of Concatenated XmoCAB

**FIGURE 1:**
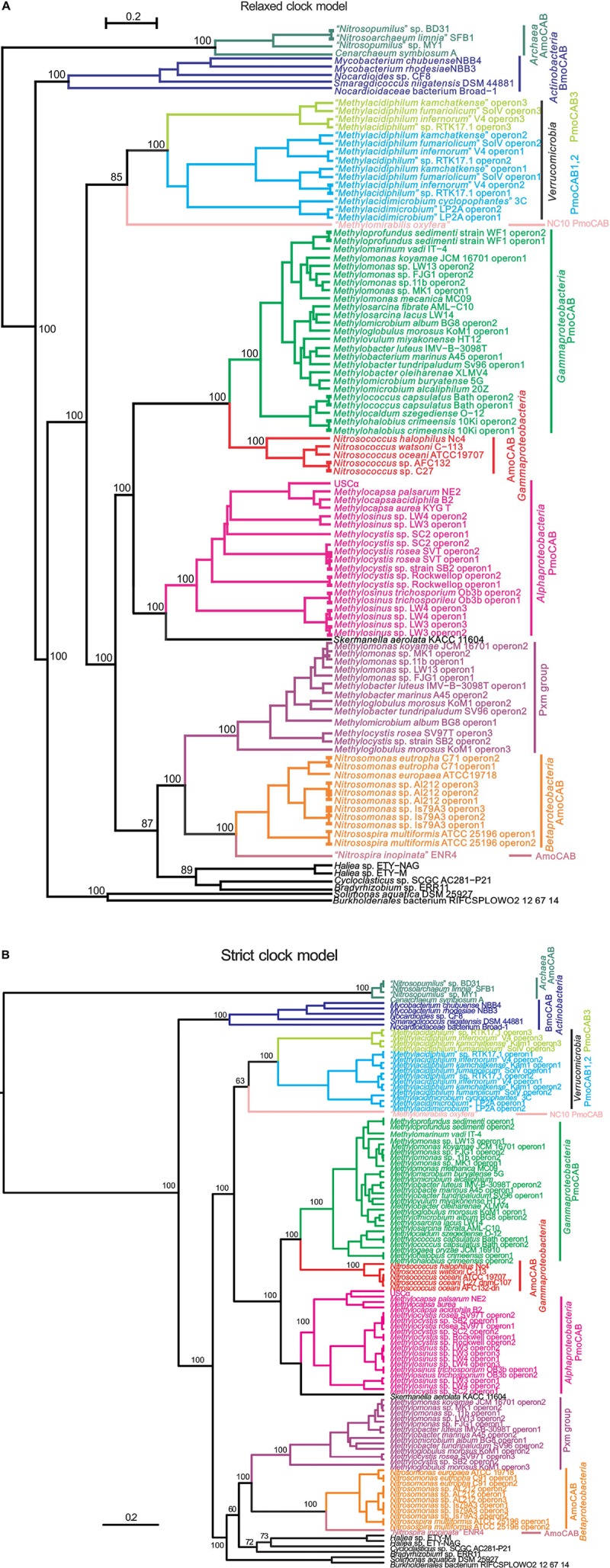
Phylogenetic trees based on concatenated inferred XmoCAB sequences (minimum 910 amino acids). The tree was constructed using Bayesian analysis employing: **(A)** a gamma site heterogeneity model with four gamma categories with a relaxed clock log normal model and **(B)** a Blosum62 substitution model with gamma site heterogeneity model under a strict clock. Node values are based on 10,000,000 iterations, minus a burn-in of 20% of the total. The scale bar represents 0.2 changes per amino acid position. Colors indicate coherent functional and taxonomic groups. The protein accession numbers for the operons are given in Supplementary Table S3.

Highly resolved phylogenies of concatenated inferred XmoCAB sequences were constructed via Bayesian inference methods (strict clock vs. relaxed clock log-normal model) (Figure 1), as well as ML and NJ methods (Supplementary Figures S1A,B). We constructed phylogenies of XmoCAB both with and without thaumarcheotal XmoCAB sequences included, since a large number of sequence gaps/insertions when comparing bacterial and thaumarchaeotal sequences reduce the amount of information available for phylogenetic calculations. Results were similar in each case.

The different construction methods largely agreed, with AMO from *Thaumarchaeota* the most distant group (and a logical outgroup based on total evolutionary distance), and a highly supported primary node separating the AMO of *Thaumarchaeota*, the BMO of *Actinobacteria*, and all others. All constructions generally agreed upon at least 10 well-supported monophyletic clusters corresponding to a combination of function (e.g., pMMO vs. AMO) and taxonomic affiliation. These clusters are summarized by the different colors in Figure 1. Support values for these taxonomic/functional clusters are very high in all constructions, reaching 100% posterior probabilities in Bayesian analyses. Relationships across these groups were slightly variable in the different constructions. Most notable was a variable placement of the *Verrucomicrobia* and NC10. In the Bayesian relaxed clock model and the NJ tree these methanotroph groups were monophyletic with the proteobacterial methanotrophs, in the Bayesian strict clock model and the ML model they were not.

Most of the groups indicated in Figure 1 also represent coherent monophyletic clusters based on 16S rRNA phylogeny, but they are separated by vast numbers of species that do not have *xmoCAB* genes in their genomes. The most parsimonious scenario for the distribution of *xmoCAB* is therefore that a single common ancestor of each of the groups indicated in Figure 1 received *xmoCAB* via a single LGT event. Possible instances where recent LGT has disrupted coherent taxonomic groups include: (i) the *pxmABC* of the alphaproteobacterium *Methylocystis rosea*, which clusters within a group of *Gammaproteobacteria*, and (ii) several sequences of unknown function (indicated in black) belonging to *Alpha- Beta-*, and *Gamma-proteobacteria*, particularly near the base of Figure 1. However, while the sequences indicated in black are included to support tree construction, we prefer not to discuss them in detail because: (1) in most cases the function of the respective Xmo enzymes is unknown; (2) most of the branches are deeply rooted and contain a single sequence, and the exact positioning of such branches is problematic due to plesiomorphic character states, and (3) there are still too few of these sequences to draw solid conclusions. Therefore, we focus our analysis on the better known groups of methane and ammonia oxidizers.

### Phylogenetic Analyses of Individual Genes

**FIGURE 2:**
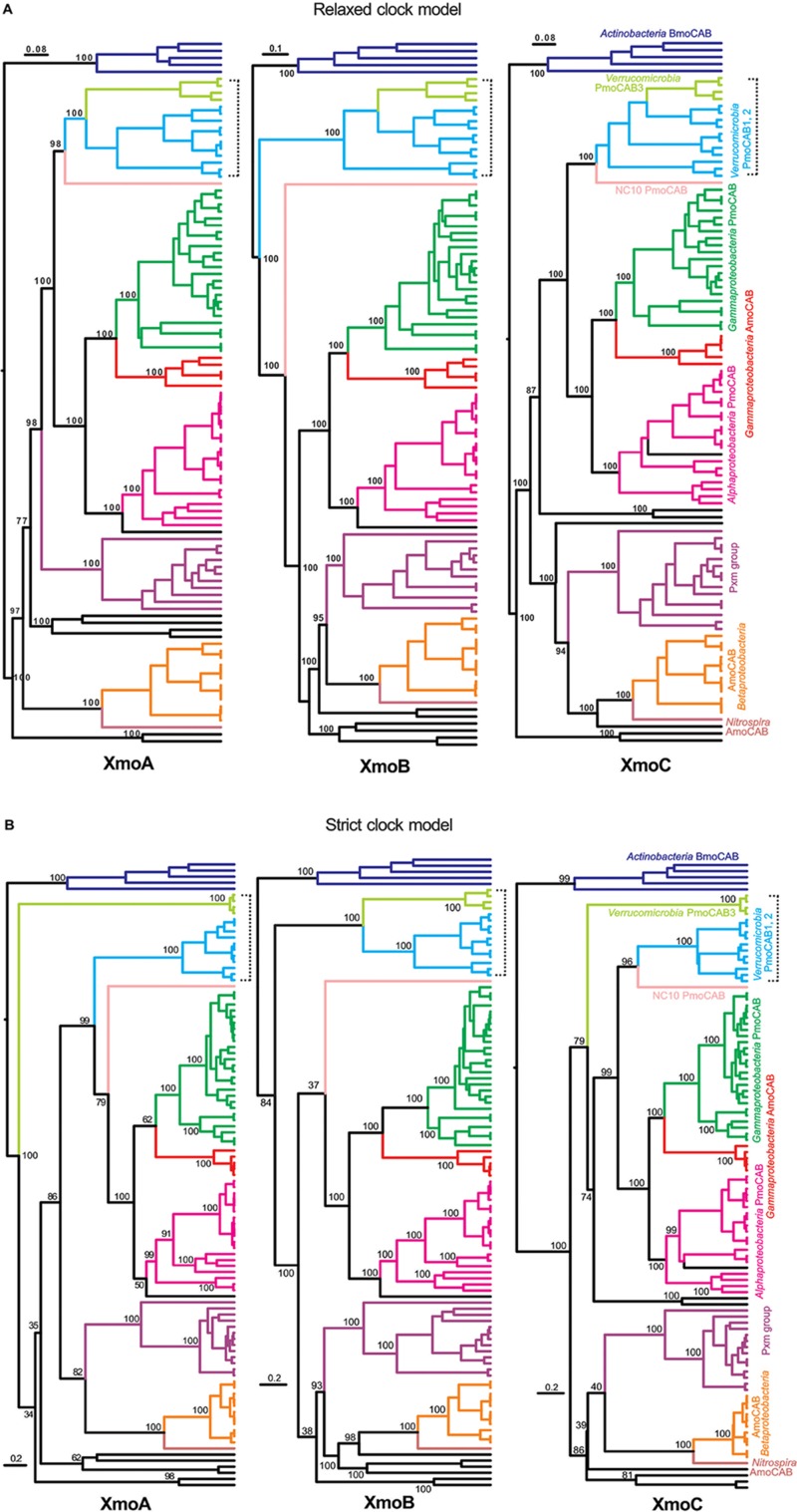
Phylogenetic trees based on inferred XmoA, B, and C sequences. Tree was constructed using Bayesian analysis employing: **(A)** a Gamma site heterogeneity model with four gamma categories with a relaxed clock log normal model and **(B)** a Blosum62 substitution model with gamma site heterogeneity model under a strict clock. Node values are based on 10,000,000 iterations after a burn-in of 20% of the total trees. The scale bars represents 0.08, 0.1, and 0.08 **(A)** and 0.2 **(B)** changes per amino acid position. Lineages are colored and labeled as in Figure 1. The bracket stresses the differences between verrucomicrobial methanotrophs across the individual trees.

Phylogenies of individual inferred XmoA, XmoB, and XmoC polypeptides were constructed using Bayesian inference (strict clock vs. relaxed clock log normal model, Figure 2), ML, and NJ methods (Supplementary Figures S2, S4A–C, respectively). When included, thaumarchaeotal sequences always formed the most distant group. However removing thaumarchaeotal sequences in some of the individual gene trees showed better support values because the large number of sequence gaps/insertions in the thaumarchaeotal genes greatly reduce the amount of information available for phylogenetic calculations. Only trees without thaumarchaeotal sequences are therefore presented. The Bayesian phylogenies are summarized in Figure 2 in a manner to stress the similarities of the three individual XmoA XmoB and XmoC trees. Generally, the same taxonomic/functional groups are identified in the individual trees as in the concatenated tree, suggesting that the three genes/polypeptides have mostly parallel evolutionary histories and are evolved or transferred as a coherent operon.

One major inconsistency in the individual trees is the placement of the three homologs found in *Verrucomicrobia*. In the Bayesian (strict molecular clock model) constructions (Figure 2B) the XmoC3 and XmoA3 homologs separate out from the respective XmoC1/XmoC2 and XmoA1/XmoA2 homologs. However this separation is highly dependent on the construction method and often not well-supported (Supplementary Figures S2A–C, S4A–C). It is not seen in the Bayesian (relaxed molecular clock model) (Figure 2A) where the all three homologs in the *Verrucomicrobia* are always monophyletic.

Another inconsistency is that for XmoB, the entire cluster of *Verrucomicrobia* is more ancestral in the tree than it is for XmoA and XmoC (Figure 2). XmoA and XmoC of *Verrucomicrobia* are monophyletic with the corresponding homologs in proteobacterial methanotrophs, but XmoB is not. The uniqueness of the XmoB phylogeny compared to XmoA and C is seen in all four methods (Bayesian strict clock, Bayesian relaxed clock, ML and NJ) (Figure 2 and Supplementary Figures S3A–C).

### Compositional LGT Detection

**FIGURE 3:**
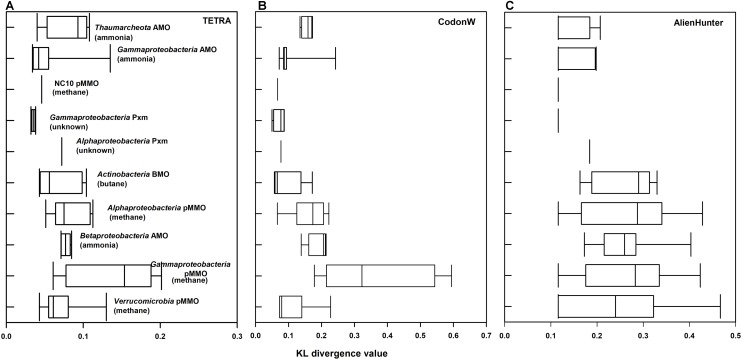
KL divergence measure of xmo*CAB* versus the entire genome in different taxonomic/functional groups of *xmoCAB*-possessing bacteria. Values on the x axes represent calculated KL values (0–1) by three different analysis: **(A)** TETRA, **(B)** CodonW, and **(C)** Alien Hunter. Larger values indicate stronger compositional differences in *xmoCAB* operons compared to their genomes and may suggest lateral gene transfer (LGT).

The KL divergences calculated with Alien Hunter, TETRA, and CodonW are summarized in Figure 3 and Supplementary Table S1. Higher values indicate greater compositional differences between the *xmoCAB* operon and host genome. Alien Hunter found evidence of significant compositional differences in most of the main clusters we identified in Figure 1. This supports the hypothesis that each group arose via an ancestral LGT event, and the composition of many *xmo* operons has not yet normalized to the composition of the host organisms.

The notable exceptions showing little or no compositional bias in the *xmo* operon were the gammaproteobacterial Pxm cluster, the gammaproteobacterial AMO (*Nitrosococcus* spp.), and the NC10 pMMO. These were below the significance threshold using Alien Hunter (Supplementary Table S1) and also showed the lowest KL values of all the groups in other compositional analyses (Figure 3). These KL values calculated using CodonW and TETRA differ absolutely from those of Alien Hunter, but generally agree on the operons with the highest compositional differences to their genomes (Figure 3). Although the acclimatization rates of foreign DNA to a genome may vary, we speculate that these three groups may represent those that have possessed the operon for the longest evolutionary time, and their ancestors were likely donors of *xmo* to the other groups.

On the other hand, several clusters of *xmoCAB* show extremely high compositional differences to their host genomes: particularly those encoding the pMMO in *Gammaproteobacteria* and the AMO in *Betaproteobacteria*, but also to a lesser extent the pMMO in *Alphaproteobacteria* and the BMO in *Actinobacteria*. These may represent more recent LGT acquisitions of *xmoCAB*. This hypothesis is supported by the analysis using IslandViewer 4, a tool for detection and visualization of genomic islands (Bertelli et al., 2017), which shows that *xmoCAB* operons in some betaproteobacterial nitrifiers, actinobacterial butane oxidizers, and gammaproteobacterial methanotrophs can still be detected on genomic islands likely transferred from a foreign genome (Supplementary Table S1).

### Mutation Rate Analyses

Based on compositional analysis (Supplementary Table S1), *Methylacidiphilum* sp. *xmoCAB3* operons have high KL values for Alien Hunter (0.47), TETRA (0.13), and CodonW (0.23) compared to the other two operons in the *Verrucomicrobia*. This suggests that they are compositionally foreign. They are also placed variably in different phylogenies.

Longer branch lengths to the *xmoCAB3* genes were also observed in some phylogenies. Long branch lengths are associated with accelerated mutation rates. In order to quantify this we used Bayesian inference phylogeny with a relaxed clock log-normal model to estimate the evolutionary rate at each branch in the phylogenetic trees (Bouckaert et al., 2014; Ho and Duchene, 2014). The evolutionary rate of verrucomicrobial *pmoCAB3* genes was higher than the evolutionary rates of the *pmoCAB1 and pmoCAB2* operon copies, with rates of 2.66, 2.64, and 3.46 for XmoA, XmoB, and XmoC phylogenies respectively (Supplementary Figures S5A–C). Evolutionary rates were mostly below 0.99 for most of the other lineages. This high rate suggests that verrucomicrobial *pmoCAB* operon three genes are mutating at high rate.

### Assessing *xmoA* Primer Sets

We tested specificity of different primers that have been used to amplify *xmoA* genes from environmental DNA extracts. These included several “universal” and group-specific primers for methanotroph *pmoA* genes. The results are summarized in Supplementary Table S2. The commonly used primer sets for methanotrophs 189f/682r (Holmes et al., 1995) and 189f/661 (Costello and Lidstrom, 1999) both have apparent biases and may miss many species, not only the exotic newly described methanotrophs such as the *Verrucomicrobia* and NC10, but also some proteobacteria like *Methylocapsa*. Recently, the 189f forward primer in combination with newly developed reverse primer HD616 was used to amplify CuMMO genes with high coverage (Wang et al., 2017). The HD616 primer is more universal than any other primer tested in our database (Supplementary Table S2), but the large number of primer degeneracies may result in non-specific amplifications. However, the primer does not cover specific groups like the methanotrophic USCγ group, and thaumarcheotal *amoA* genes (Wang et al., 2017).

## Discussion

Methane and ammonia oxidizing microorganisms are well-studied in different environments because of their importance in biogeochemical cycling of carbon and nitrogen. Studies based on 16S rRNA genes and functional marker genes like *amoA* and *pmoA* have been used to interpret their diversity and abundance in the environment (Knief, 2015). By using an extensive copper monooxygenase-encoding gene database from fully sequenced genomes, we constructed robust CuMMO phylogenies and performed comparative genomics to elucidate the phylogenetic history of the encoding genes.

All phylogenies showed at least 10 distinct groups based on a combination of substrate and taxonomy. Phylogenies of individual XmoA, B, and C products also showed similar patterns (with the exception of the *Verrucomicrobia*, discussed below). This suggests that the operon has evolved and been transferred primarily as a single unit. The evolutionary history of Xmo is therefore a mixture of functional evolution and a few LGT events of the entire operon, at minimum one event to each distinct group shown in Figure 1. Although to some extent CuMMOs can each oxidize all the relevant substrates (ammonia, methane, short chain alkanes and alkenes), they tend to prefer one substrate, indicating specialization to different substrates (Bedard and Knowles, 1989). The integration of CuMMOs into catabolic pathways targeting particular substrates has occurred multiple times. For example, in all phylogenies, the AMOs of *Thaumarchaeota, Betaproteobacteria*, and *Gammaproteobacteria* formed highly supported, distinct clusters, indicating that integration of CuMMO enzymes into an ammonia oxidizing metabolism occurred multiple times. The most parsimonious scenario based on the Bayesian tree (ignoring the Xmo’s of unknown function in Figure 1, and assuming Pxm is a methane monooxygenase) begins with either a pMMO or AMO ancestor followed by six changes of function.

Despite the coherent functional/taxonomic groups, there is no overriding structuring of the tree based on either function or taxonomy alone. Taxonomically, for example, *Gammaproteobacteria* clusters are scattered about the phylogenetic tree. Functionally, AMOs of *Thaumarchaeota, Betaproteobacteria*, and *Gammaproteobacteria* each form coherent, highly supported clusters, but these three do not together form a monophyletic group, indicating that incorporation of ammonia-specific CuMMO into metabolic pipelines capable of utilizing toxic hydroxylamine for catabolic gain (Simon and Klotz, 2013) has evolved multiple times. Individual pMMO clusters of *Alphaproteobacteria, Verrucomicrobia*, NC10, and *Gammaproteobacteria* methanotrophs can be discerned. These four groups all cluster closely together, however methanotrophy as a function is paraphyletic, with a group of ammonia-oxidizers (*Nitrosococcus* spp.) and the non-methanotrophic *Skermanella* nested within the larger group of methanotrophs. The *pxmABC* homologs of *pmoCAB* in methanotrophs are also not monophyletic with the known *pmoCAB* operons. Experiments with *M. denitrificans* FJG1 suggested that Pxm is an alternate methane monooxygenase that cells express particularly under microoxic conditions (Kits et al., 2015b), but its function is still not completely clear. Nevertheless, if it indeed acts as a methane monooxygenase, this pattern indicates the adaptation of CuMMOs to catabolizing methane also evolved multiple times.

### Verrucomicrobia

The major inconsistencies in different phylogenetic constructions (both in the concatenated trees and in the individual gene trees) were in the placement of the *Verrucomicrobia*. *Verrucomicrobia* methanotrophs contain up to three homologous but distinct *pmoCAB* operons, with the most divergent of these usually named *pmoCAB3* (Op den Camp et al., 2009). Some constructions showed the three operons of *Verrucomicrobia* to be polyphyletic, with XmoC3 and XmoA3 separate from the corresponding XmoC1/2 and XmoA1/2 (Figure 2). However, this was most evident in construction methods that are easily violated by variable mutation rates in different lineages (strict-clock Bayesian and NJ trees). When the clock assumption was relaxed in ML or Bayesian relaxed clock models, the three verrucomicrobial homologs were always monophyletic (Figure 2 and Supplementary Figures S2B, S3B, S4B). Long branch lengths to the *xmoCAB3* genes were observed in most phylogenies, suggesting that an accelerated mutation rate of this third operon, which probably made some phylogenetic constructions inconsistent. A Bayesian estimation of evolutionary rates suggested that the *xmoCAB3* is indeed evolving faster than other lineages in the phylogenetic tree. We therefore propose that the three operons in the *Verrucomicrobia* are monophyletic and have arisen from two lineage-specific duplications, but that operon 3 is under accelerated, relaxed selection. This is consistent with expression studies suggesting that this operon is only weakly expressed under methanotrophic growth conditions (Erikstad et al., 2012; Khadem et al., 2012; Anvar et al., 2014).

The other oddity regarding the *Verrucomicrobia* is that their XmoB gene shows a different phylogeny than XmoA and XmoC (Figure 2). In XmoA and XmoC trees, all methanotrophs including the *Proteobacteria, Verrucomicrobia*, and NC10 form a single cluster with only the *Nitrosococcus* AMO group as a nested group. However, in the XmoB trees the methanotrophs are more polyphyletic. The proteobacterial methanotrophs, NC10 and *Nitrosococcus* remain monophyletic in the XmoB tree, but the *Verrucomicrobia* are not monophyletic with them, instead branching nearer the base on the tree. This pattern is consistently seen regardless of the construction method used. It strongly suggests a distinct evolutionary history of XmoB compared to XmoA and XmoC in this phylum. A possible evolutionary scenario to explain this pattern is that the pMMO evolved only once, as indicated by XmoA and XmoC (and XmoCAB) phylogenies, but that the *Verrucomicrobia* at one time exchanged their *xmoB* gene(s) for that from another (unknown) donor organism. Crystal structure of pMMOs from *Methylococcus capsulatus, Methylosinus trichosporium* OB3b, *Methylocystis* sp. strain M and *Methylocystis* sp. Rockwell all predict a copper binding site at the N-terminus of the PmoB subunit (Sirajuddin and Rosenzweig, 2015). This copper binding site is conserved in all methanotrophs except for the *Verrucomicrobia* (Op den Camp et al., 2009). It is possible that the *Verrucomicrobia* have adopted a more distant PmoB to alter the metal profile of their enzymes (Culpepper and Rosenzweig, 2012).

### Lateral Gene Transfer

**FIGURE 4:**
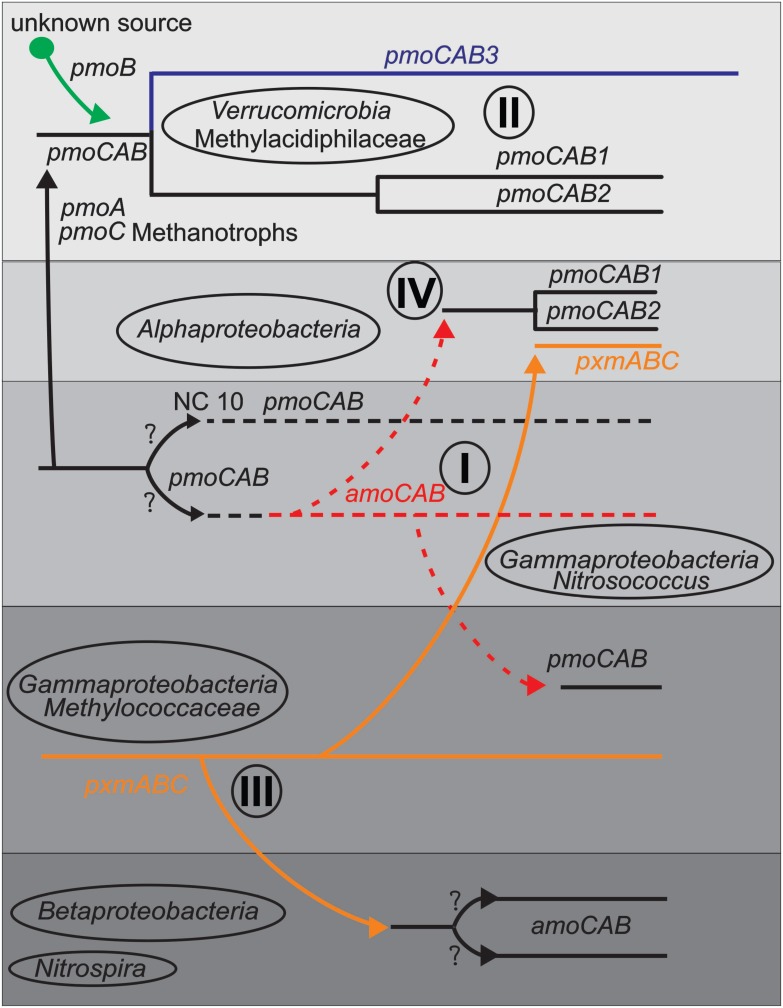
Schematic diagram indicating some predicted LGT events (arrows) between taxa or lineages based on phylogeny and compositional analysis items I-IV discussed in the text. Shaded boxes represent taxa and lines represents gene lineages.

Detecting LGT generally relies on phylogenetic incongruence between different gene trees (Delwiche and Palmer, 1996; Daubin et al., 2003; Lerat et al., 2003; Beiko et al., 2005; Adato et al., 2015), and on the compositional biases of genes or operons compared to genomes (Garcia-Vallve et al., 2000; Karlin, 2001; Teeling et al., 2004a). We used both phylogenetic interference and compositional analysis methods to detect possible LGT events. Compositional analysis showed evidence of extensive LGTs, while phylogenies demonstrated that these were limited to few (minimum 10) total events, followed by diversification of functional/taxonomic groups. From the phylogenetic and compositional analyses, we postulated several possible routes of LGT into different taxa and lineages, summarized in Figure 4. We restrict our speculation to clusters containing many sequences, and therefore prefer not to speculate greatly on the NC10 and *Nitrospira*, although the history of LGT from or to these groups would also be illuminating.

(I) The *Nitrosococcus amoCAB* cluster is nested within a large cluster of methanotrophic *pmoCAB*s, but compositional analysis indicates that it is more native to its genomes than the *pmoCAB*s are to the methanotrophs. *pmoCAB* operons in *Alphaproteobacteria* and *Gammaproteobacteria* each show strong evidence of LGT. We therefore propose that the ancestor of the gammaproteobacterial *Nitrosococcus* group was a methanotroph with a *pmoCAB* operon. This *Nitrosococcus* lineage first transferred its *pmoCAB* genes to *Alphaproteobacteria* methanotrophs and then later to the gammaproteobacterial methanotrophs. This fits with recent analyses showing that some gammaproteobacterial nitrifiers: specifically *Nitrosococcus wardiae* (Wang et al., 2016) and *Nitrosococcus halophilus* (Campbell et al., 2011a), encode a complete methane-oxidation pipeline, including methanol dehydrogenase, a tetrahydromethanopterin-based formaldehyde oxidation pathway, and formate dehydrogenase (Chistoserdova, 2016; Wang, 2016). These findings may indicate that ancestors of extant *Nitrosococcus* once functioned primarily as methanotrophs. The AMO from *Nitrosococcus oceani* also does not exhibit substrate preference for ammonia or methane (Lontoh et al., 2000).

This pattern of lateral transfer of genes implicated in ammonia catabolism from ancestors of *Nitrosococcus* to the ancestors of extant gammaproteobacterial methanotrophs has also been proposed based on an analysis of the gene cluster encoding hydroxylamine dehydrogenase (Wang, 2016). Hydroxylamine dehydrogenase detoxifies and extracts catabolic electrons from hydroxylamine, the product of ammonia oxidation by CuMMO, and is co-expressed in ammonia-catabolic bacteria with the cytochrome *c*-based conduit that channels the extracted electrons from the periplasm into the Quinone-pool (Klotz and Stein, 2011; Simon and Klotz, 2013). Interestingly, the hydroxylamine detox capacity has been retained only in methanotrophs that are exposed to fluctuating ammonium concentrations in the environment (Nyerges and Stein, 2009; Nyerges et al., 2010; Campbell et al., 2011b; Stein and Klotz, 2011) but lost in those that typically exist in low ammonium environments (Tamas et al., 2014).

(II) The “*Methylacidiphilum*” group possesses two similar (*pmoCAB1* and *pmoCAB2*) and one divergent *pmoCAB3* operon. All are monophyletic, but the phylogeny of the *pmoB* differs from that of *pmoC* and *pmoA.* We propose that the ancestor of “*Methylacidiphilum*” obtained *pmoCAB* genes from an ancestral methanotroph (perhaps the NC10 lineage or the *Nitrosococcus* ancestor), but replaced that original *pmoB* gene with an *xmoB* gene from another, unknown source. The three operons were created by two successive lineage specific duplications. High evolutionary rates are seen along the verrucomicrobial *pmoCAB3* operon, and make some phylogenetic constructions inconsistent.
(III) In the *Gammaproteobacteria* methanotrophs, the *pxmABC* operon is compositionally indistinguishable from the genomes. Their *pmoCAB* operons, on the other hand, are compositionally very distinct from most genomes. This suggests that these bacteria first possessed the *pxmABC* operon, and only later obtained the *pmoCAB* operon via LGT (from the ancestor of *Nitrosococcus*). It has been speculated that gammaproteobacterial *pxm* genes were horizontally transferred (Tavormina et al., 2011); however, above analyses support the opposite evolutionary scenario. The *Gammaproteobacteria pxmABC* operon may also have been transferred to some *Alphaproteobacteria* methanotrophs, and to the *Betaproteobacteria* nitrifiers.
(IV) As previously proposed (Tamas et al., 2014), a methylotrophic ancestor of all alphaproteobacterial methanotrophs in the families *Methylocystaceae* and *Beijerinckiaceae* obtained pMMO via a single LGT event from a gammaproteobacterial ancestor. This operon underwent a duplication and divergence into two lineages in the *Methylocystaceae*, and was lost in many *Beijerinckiaceae*. A second transfer event of the *pxmABC* operon from the *Gammaproteobacteria* has also occurred.

We stress that the evolution of a methanotrophic or ammonia-oxidizing phenotype is more complicated than the simple acquisition of a CuMMO. The downstream metabolic machinery to detoxify toxic alcohol and aldehyde intermediates such as methanol, formaldehyde, and hydroxylamine, while simultaneously feeding catabolic electrons into the Q-pool, are as critical as the monooxygenase step (Klotz and Stein, 2008, 2011; Simon and Klotz, 2013). Hence, the acquisition of the genetic basis for a functional CuMMO (*xmoCAB*) has to be preceded by the existence of genes that encode detoxification and electron extraction modules paired with a respective quinone-reactive protein (QRP) in order to create an efficient electron-flow pipeline that will integrate the CuMMO to the genome. Hence, it has been proposed for example that the original alphaproteobacterium that obtained *pmoCAB* via LGT must have been a methylotroph, a proposition strongly supported by phylogenetic examination of methylotrophy genes (Tamas et al., 2014). In this study we have concentrated on the critical first step of the process, and elucidated some of the steps in the evolution, transfer, duplication, and adaptation of CuMMOs.

## Author Contributions

RK, LC, LW, CL, MK, and PD designed the experiments. RK conducted the majority of work including phylogenetic and compositional analysis of genomes. LW, CL, and MK generated unpublished *Nitrosococcus* genome sequences. LC initiated compositional analysis work including TETRA, CodonW and Alien Hunter and RK continued further analysis. RK and PD wrote the manuscript. All authors provided input on the final manuscript.

## Conflict of Interest Statement

The authors declare that the research was conducted in the absence of any commercial or financial relationships that could be construed as a potential conflict of interest.

## Footnotes

1 http://genome.jgi.doe.gov/

2 http://www.ncbi.nlm.nih.gov/nucleotide

3 http://rast.theseed.org/FIG/rast.cgi

4 http://www.megx.net/tetra/index.html

5 http://www.cbs.dtu.dk/services/FeatureExtract/

6 http://mobyle.pasteur.fr/cgi-bin/portal.py#forms::CodonW
