# Supplementary material for: Evolutionary History of Copper Membrane Monooxygenases
Source: Front Microbiol. 2018 Oct 29;9:2493. doi: 10.3389/fmicb.2018.02493 (PMC6215863; doi:10.3389/fmicb.2018.02493)
Supplement: Supplementary file 1 [file Image_1.pdf]

Supplementary Material

Phylogenetic history of Copper Membrane Monooxygenases

Roshan Khadka[1], Lindsay Clothier[1], Lin Wang[2†], Chee Kent Lim[2¥], Martin G. Klotz[3,4], and Peter F. Dunfield[1*]

[1] Department of Biological Sciences, University of Calgary, 2500 University Drive NW, Calgary, Alberta, Canada, T2N 1N4

[2] Department of Biological Sciences, University of North Carolina, Charlotte, NC, United States

[3] School of Molecular Biosciences, College of Veterinary Medicine, Washington State University, Richland, WA, United States; ORCID: 0000-0002-1783-375X

[4] State Key Laboratory of Marine Environmental Science, Institute of Marine Microbes and Ecospheres, College of Ocean and Earth Sciences, Xiamen University, Xiamen, China

* Correspondence: PF Dunfield Tel: 403-220-2469 Fax: 403-289-9311 E-mail: pfdunfie@ucalgary.ca

[†] Present address: Center for Microbial Pathogenesis, Institute for Biomedical Sciences, Georgia State University, Atlanta, GA 30303, United States

[¥] Present address: School of Energy & Environment, City University of Hong Kong, Kowloon, Hong Kong, SAR

* **Correspondence**: PF Dunfield Tel: 403-220-2469 Fax: 403-289-9311 E-mail:

pfdunfie@ucalgary.ca

Supplementary Figures and Tables

Supplementary Figures

Supplementary Figure S1A. Maximum-likelihood XmoCAB based phylogeny of a Cu-monooxygenase. The tree was constructed using Seaviw 4.4.12 (Gouy et al., 2010) employing an LG model (Le and Gascuel, 2008) (100 iterations). Node values are based on 100 bootstrap replicates. The scale bar represents 0.2 change per amino acid position. Colours indicate coherent functional and taxonomic groups.

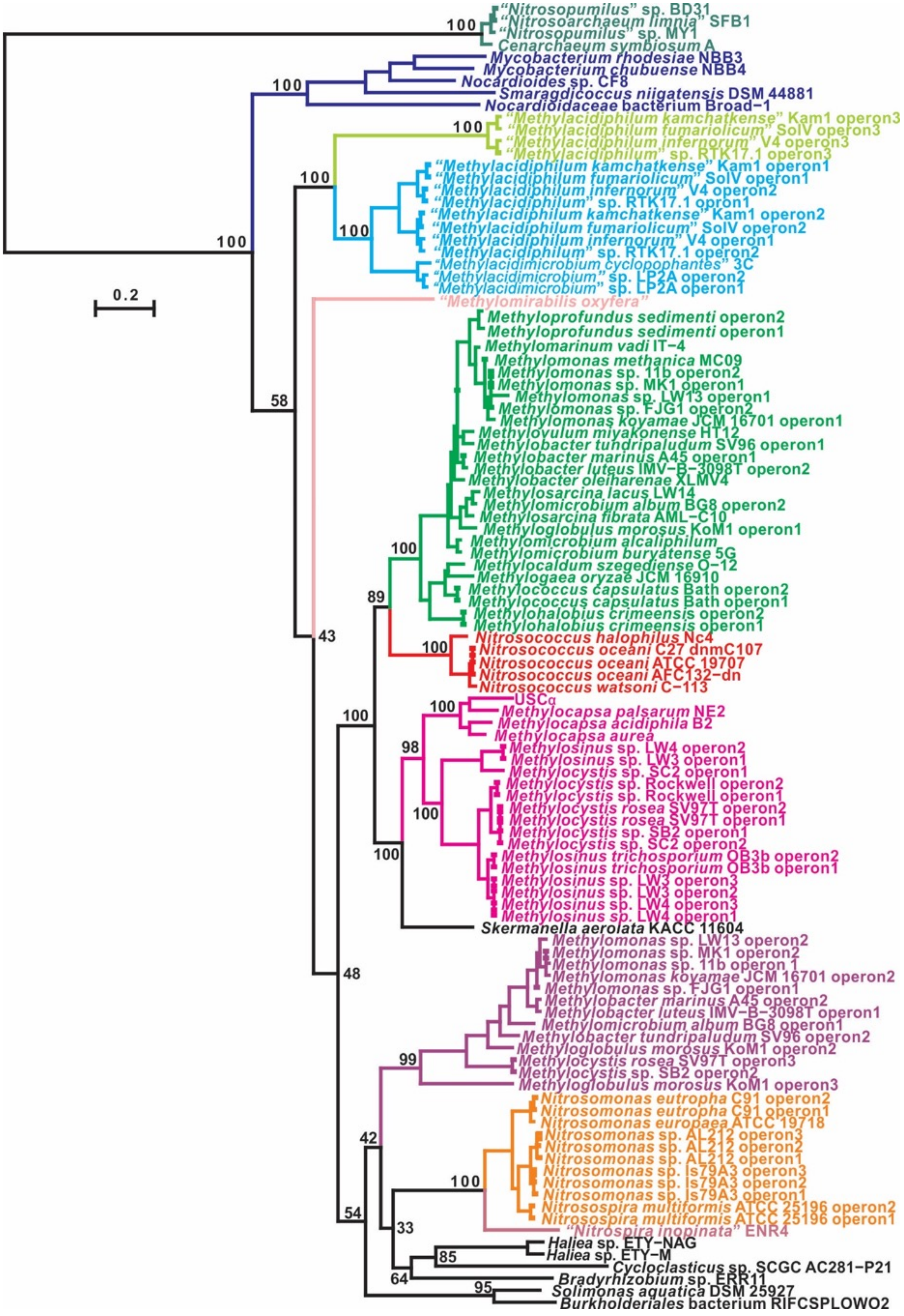

"Nitrosopumilus" sp. BD31
"Nitrosoarchaeum limnia" SFB1
"Nitrosopumilus" sp. MY1
Cenarchaeum symbiosum A
Mycobacterium rhodesiae NBB3
Mycobacterium chubuense NBB4
Nocardioides sp. CF8
Smaragdicoccus niigatensis DSM 44881
Nocardioidaceae bacterium Broad−1
"Methylacidiphilum kamchatkense" Kam1 operon3
"Methylacidiphilum fumariolicum" SolV operon3
"Methylacidiphilum infernorum" V4 operon3
"Methylacidiphilum" sp. RTK17.1 operon3
"Methylacidiphilum kamchatkense" Kam1 operon1
"Methylacidiphilum fumariolicum" SolV operon1
"Methylacidiphilum infernorum" V4 operon2
"Methylacidiphilum" sp. RTK17.1 opron1
"Methylacidiphilum kamchatkense" Kam1 operon2
"Methylacidiphilum fumariolicum" SolV operon2
"Methylacidiphilum infernorum" V4 operon1
"Methylacidiphilum" sp. RTK17.1 operon2
"Methylacidimicrobium cyclopophantes" 3C
"Methylacidimicrobium" sp. LP2A operon2
"Methylacidimicrobium" sp. LP2A operon1
0.2
"Methylomirabilis oxyfera"
Methyloprofundus sedimenti operon2
Methyloprofundus sedimenti operon1
Methylomarinum vadi IT−4
Methylomonas methanica MC09
Methylomonas sp. 11b operon2
Methylomonas sp. MK1 operon1
Methylomonas sp. LW13 operon1
Methylomonas sp. FJG1 operon2
Methylomonas koyamae JCM 16701 operon1
Methylovulum miyakonense HT12
Methylobacter tundripaludum SV96 operon1
Methylobacter marinus A45 operon1
Methylobacter luteus IMV−B−3098T operon2
Methylobacter oleiharenae XLMV4
Methylosarcina lacus LW14
Methylomicrobium album BG8 operon2
Methylosarcina fibrata AML−C10
Methyloglobulus morosus KoM1 operon1
Methylomicrobium alcaliphilum
Methylomicrobium buryatense 5G
Methylocaldum szegediense O−12
Methylogaea oryzae JCM 16910
Methylococcus capsulatus Bath operon2
Methylococcus capsulatus Bath operon1
Methylohalobius crimeensis operon2
Methylohalobius crimeensis operon1
Nitrosococcus halophilus Nc4
Nitrosococcus oceani C27 dnmC107
Nitrosococcus oceani ATCC 19707
Nitrosococcus oceani AFC132−dn
Nitrosococcus watsoni C−113
USCα
Methylocapsa palsarum NE2
Methylocapsa acidiphila B2
Methylocapsa aurea
Methylosinus sp. LW4 operon2
Methylosinus sp. LW3 operon1
Methylocystis sp. SC2 operon1
Methylocystis sp. Rockwell operon2
Methylocystis sp. Rockwell operon1
Methylocystis rosea SV97T operon2
Methylocystis rosea SV97T operon1
Methylocystis sp. SB2 operon1
Methylocystis sp. SC2 operon2
Methylosinus trichosporium OB3b operon2
Methylosinus trichosporium OB3b operon1
Methylosinus sp. LW3 operon3
Methylosinus sp. LW3 operon2
Methylosinus sp. LW4 operon3
Methylosinus sp. LW4 operon1
Skermanella aerolata KACC 11604
Methylomonas sp. LW13 operon2
Methylomonas sp. MK1 operon2
Methylomonas sp. 11b operon 1
Methylomonas koyamae JCM 16701 operon2
Methylomonas sp. FJG1 operon1
Methylobacter marinus A45 operon2
Methylobacter luteus IMV−B−3098T operon1
Methylomicrobium album BG8 operon1
Methylobacter tundripaludum SV96 operon2
Methyloglobulus morosus KoM1 operon2
Methylocystis rosea SV97T operon3
Methylocystis sp. SB2 operon2
Methyloglobulus morosus KoM1 operon3
Nitrosomonas eutropha C91 operon2
Nitrosomonas eutropha C91 operon1
Nitrosomonas europaea ATCC 19718
Nitrosomonas sp. AL212 operon3
Nitrosomonas sp. AL212 operon2
Nitrosomonas sp. AL212 operon1
Nitrosomonas sp. Is79A3 operon3
Nitrosomonas sp. Is79A3 operon2
Nitrosomonas sp. Is79A3 operon1
Nitrosospira multiformis ATCC 25196 operon2
Nitrosospira multiformis ATCC 25196 operon1
"Nitrospira inopinata" ENR4
Haliea sp. ETY−NAG
Haliea sp. ETY−M
Cycloclasticus sp. SCGC AC281−P21
Bradyrhizobium sp. ERR11
Solimonas aquatica DSM 25927
Burkholderiales bacterium RIFCSPLOWO2

Supplementary Figure S1B. Phylogenetic tree of inferred XmoCAB sequences based on a Neighbor-joining method with Poisson model constructed using Seaview 4.4.12 (Gouy et al., 2010). Node values are based on 100 bootstrap replicates. The scale bar represents 0.2 change per amino acid position. Colours indicate coherent functional and taxonomic groups.

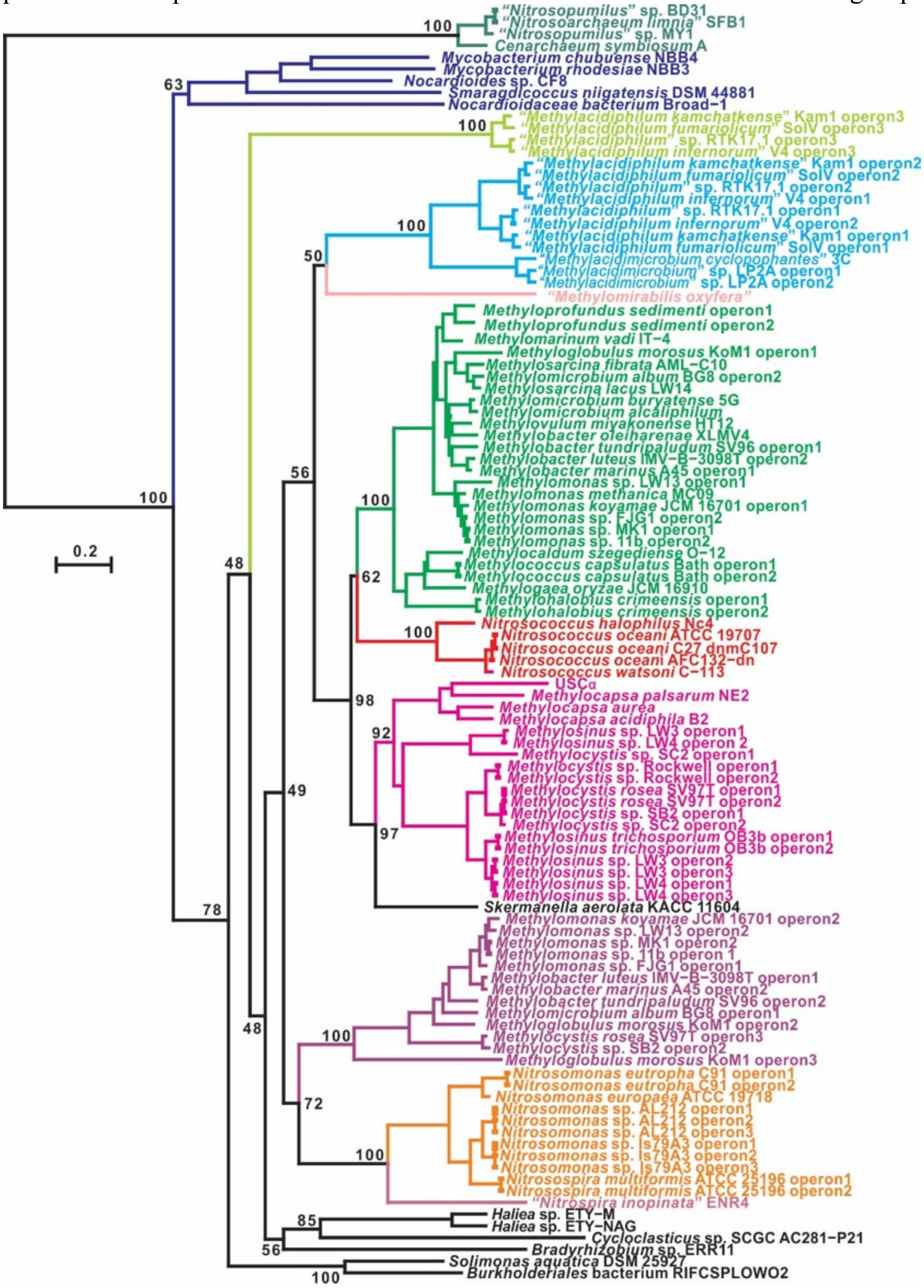

Supplementary Figure S2A. Phylogenetic tree based on inferred XmoA sequences constructed using Bayesian analysis employing a gamma site heterogeneity model with 4 gamma categories with a relaxed clock log normal model. Node values are Bayesian posterior probabilities based on 10,000,000 iterations, minus a burn-in of 20% of total. The scale bar represents 0.08 changes per amino acid position. Colours indicate coherent functional and taxonomic groups.

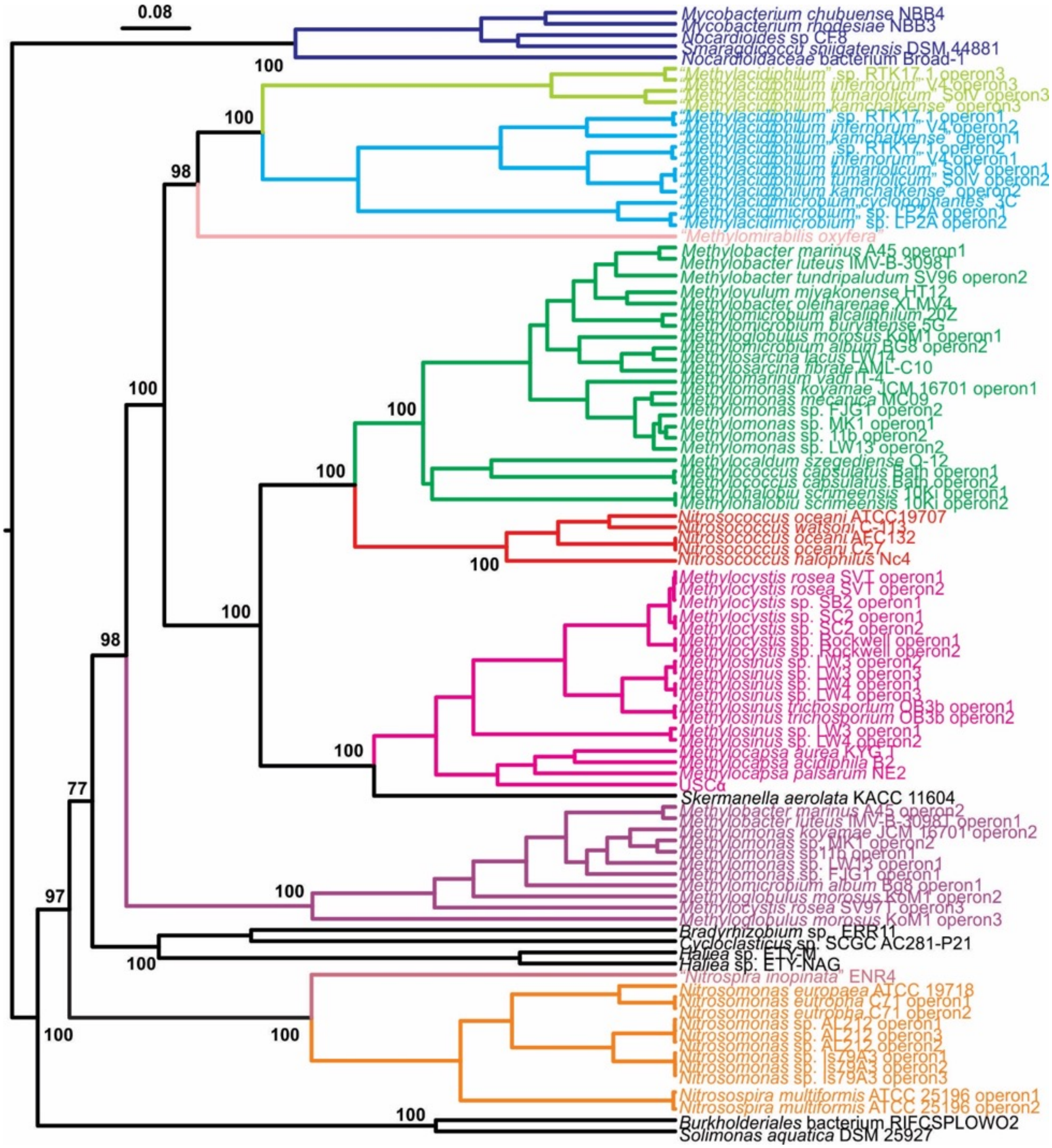

Supplementary Figure S2B. Phylogenetic tree based on inferred XmoA sequences constructed using Maximum-likelihood. The tree was constructed using Seaview 4.4.12 (Gouy et al., 2010), employing an LG model (Le and Gascuel, 2008) (100 iterations). Node values are based on 100 bootstrap replicates. The scale bar represents 0.2 changes per amino acid position. Colours indicate coherent functional and taxonomic groups.

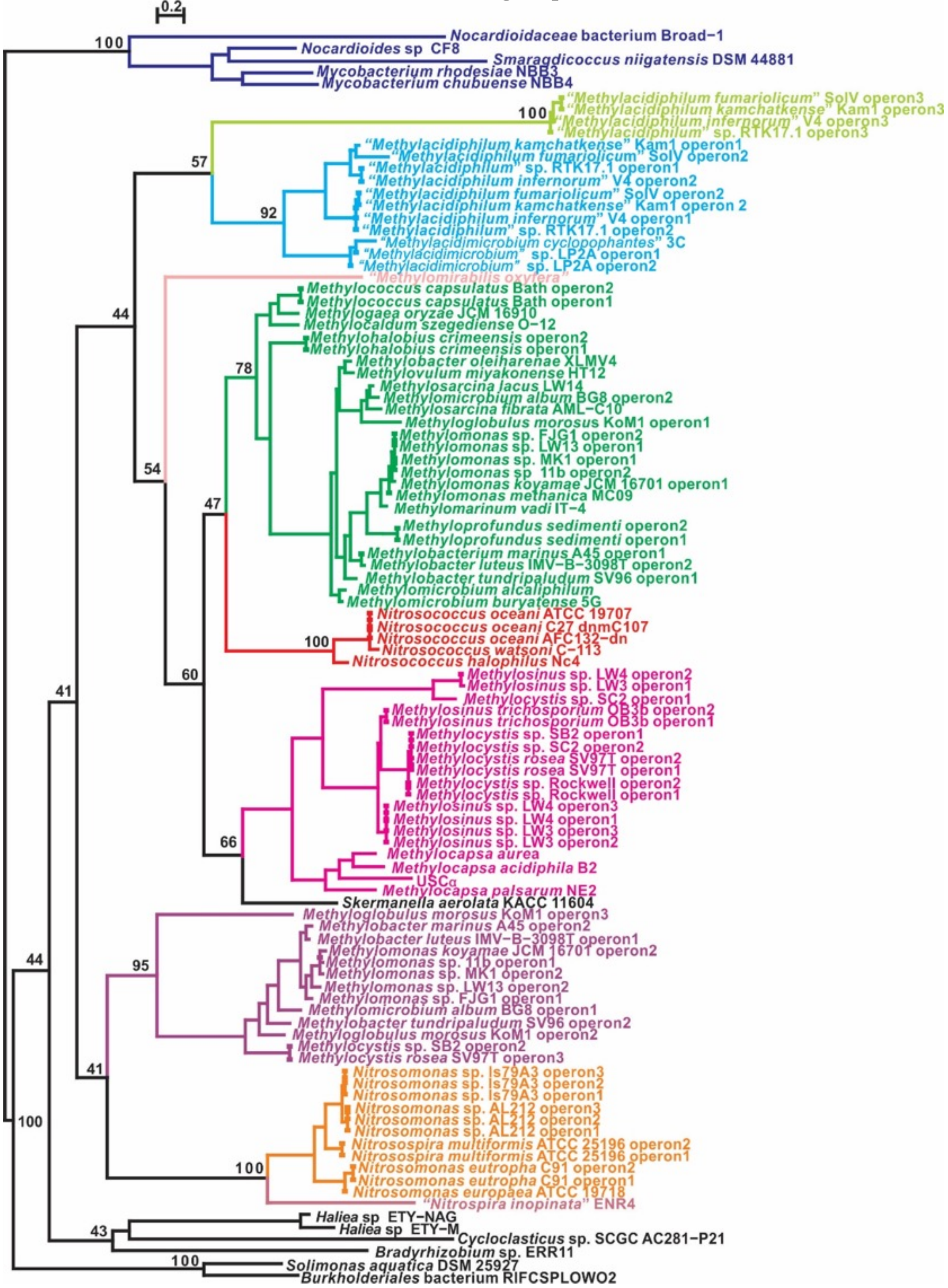

Supplementary Figure S2C. Phylogenetic tree based on inferred XmoA sequences constructed using a Neighbor-joining method with Poisson model using Seaview 4.4.12 (Gouy et al., 2010). Node values are based on 100 bootstrap replicates. The scale bar represents 0.2 changes per amino acid position. Colours indicate coherent functional and taxonomic groups.

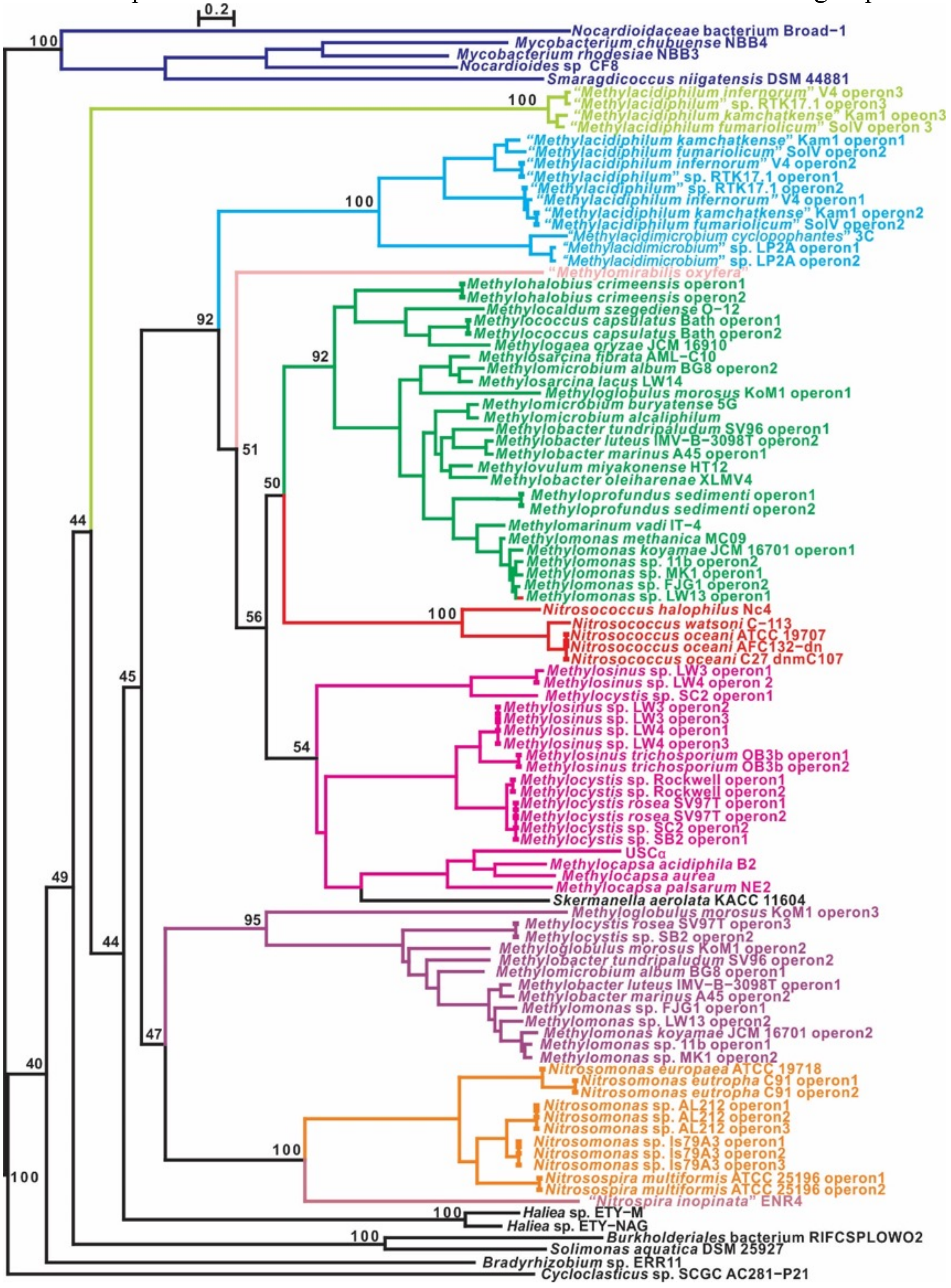

Supplementary Figure S3A. Phylogenetic tree based on inferred XmoB sequences constructed using Bayesian analysis employing a gamma site heterogeneity model with 4 gamma categories with a relaxed clock log normal model. Node value are Bayesian posterior probabilities based on 10,000,000 iterations, minus a burn-in of 20% of total. The scale bar represents 0.01 changes per amino acid position. Colours indicate coherent functional and taxonomic groups

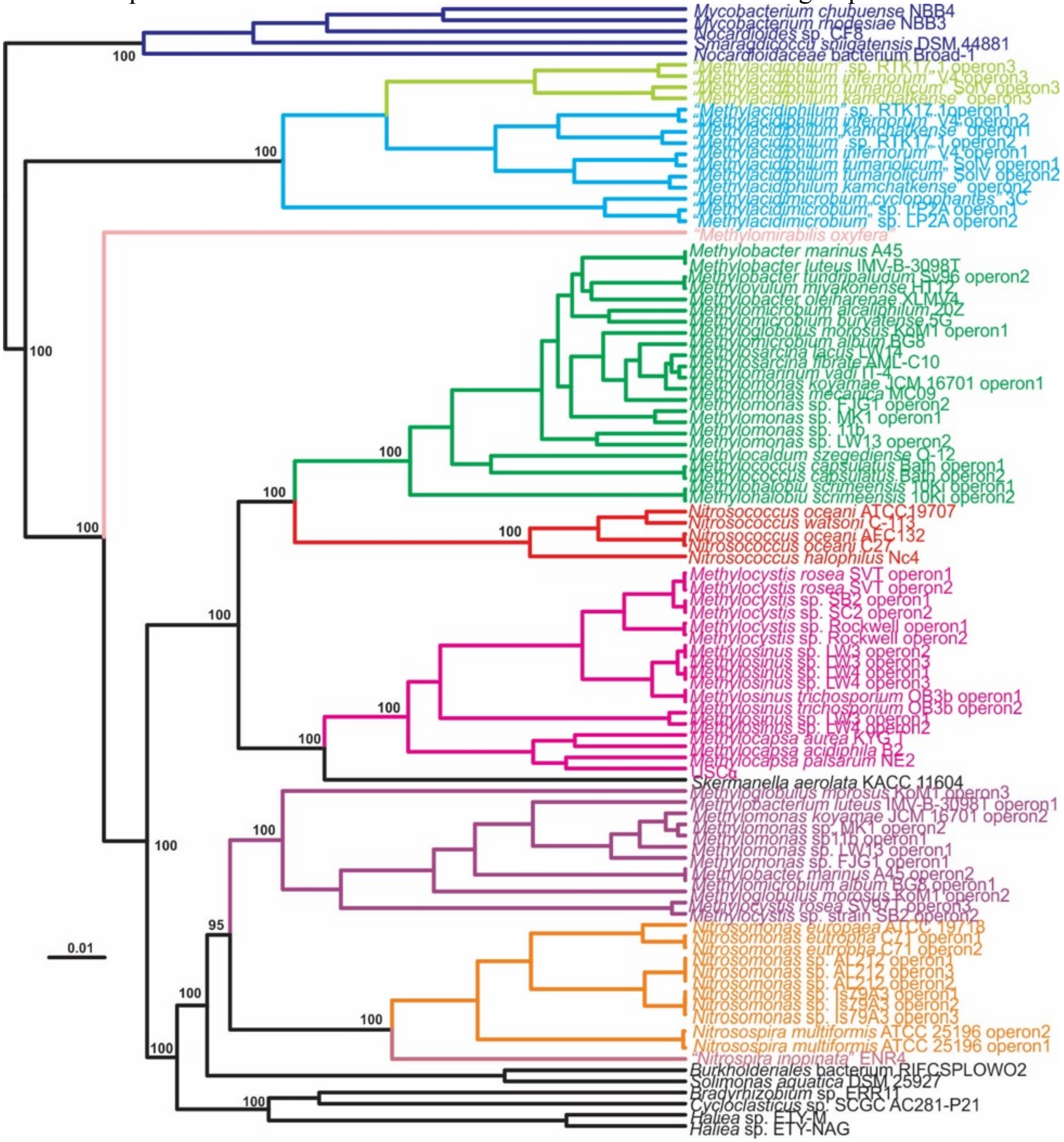

Supplementary Figure S3B. Phylogenetic tree based on inferred XmoB sequences constructed using Maximum-likelihood. The tree was constructed using Seaview 4.4.12 (Gouy et al., 2010), employing an LG model (Le and Gascuel, 2008) (100 iterations). Node values are based on 100 bootstrap replicates. The scale bar represents 0.2 changes per amino acid position. Colours indicate coherent functional and taxonomic groups.

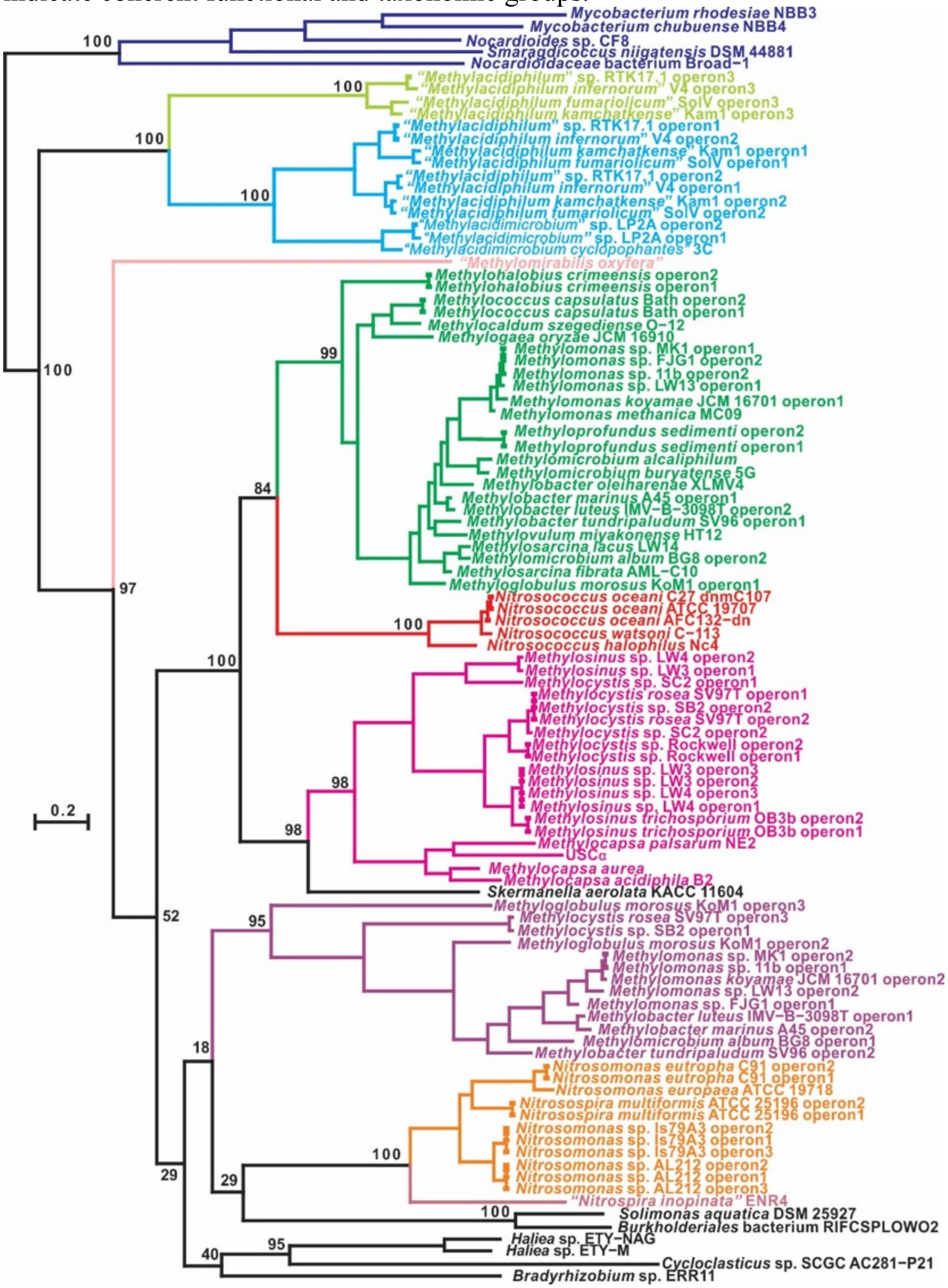

Supplementary Figure S3C: Phylogenetic tree of inferred XmoB sequences constructed using Neighbor-joining with a Poisson model. The tree was constructed using Seaview 4.4.12 (Gouy et al., 2010). Node values are based on 100 bootstrap replicates. The scale bar represents 0.2 changes per amino acid position. Colours indicate coherent functional and taxonomic groups.

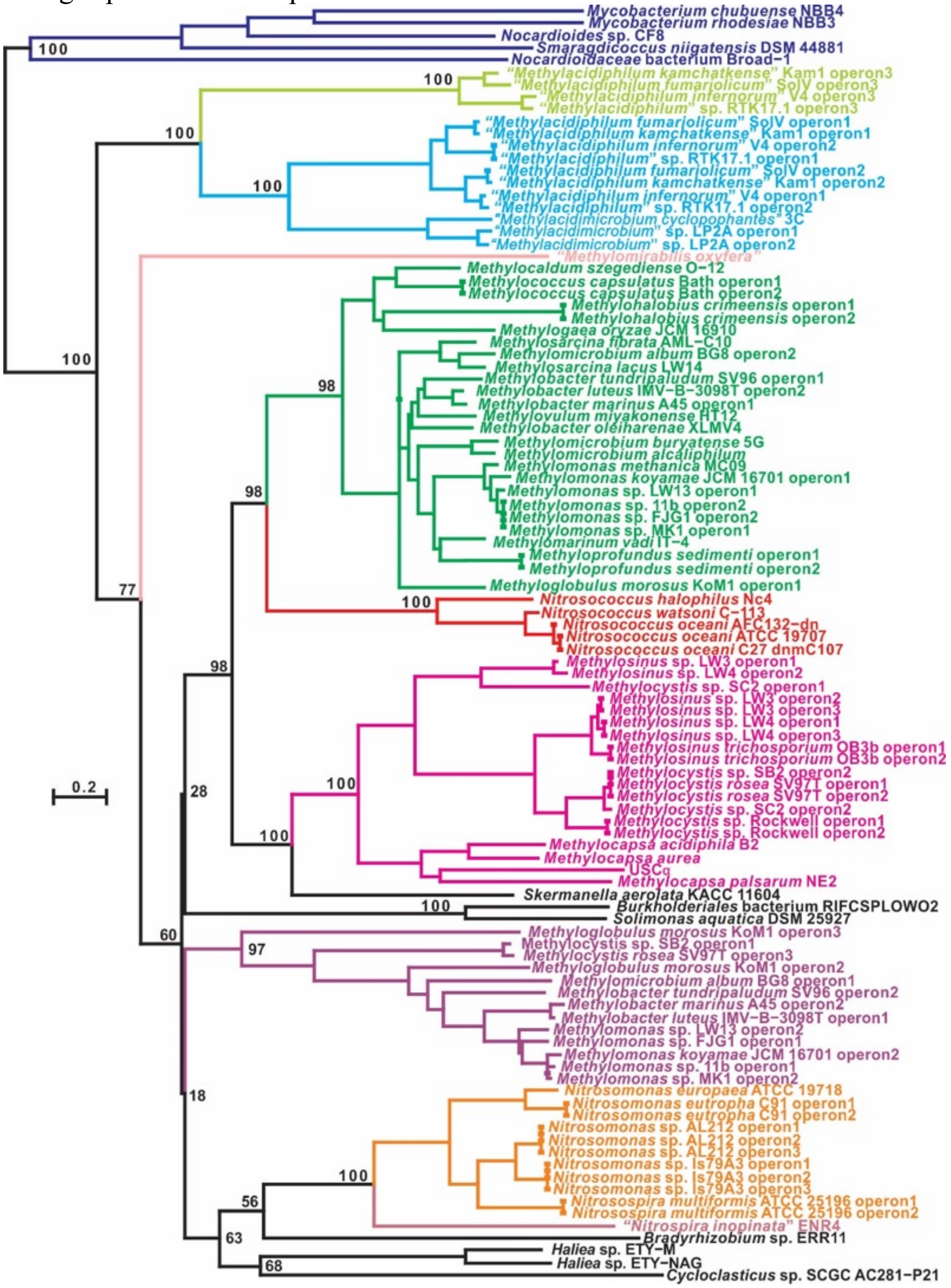

Supplementary Figure S4A. Phylogenetic tree of inferred XmoC sequences, constructed using Bayesian analysis employing a gamma site heterogeneity model with 4 gamma categories with a relaxed clock log normal model. Node value are Bayesian posterior probabilities based on 10,000,000 iterations, minus a burn-in of 20% of total. The scale bar represents 0.08 changes per amino acid position. Colours indicate coherent functional and taxonomic groups.

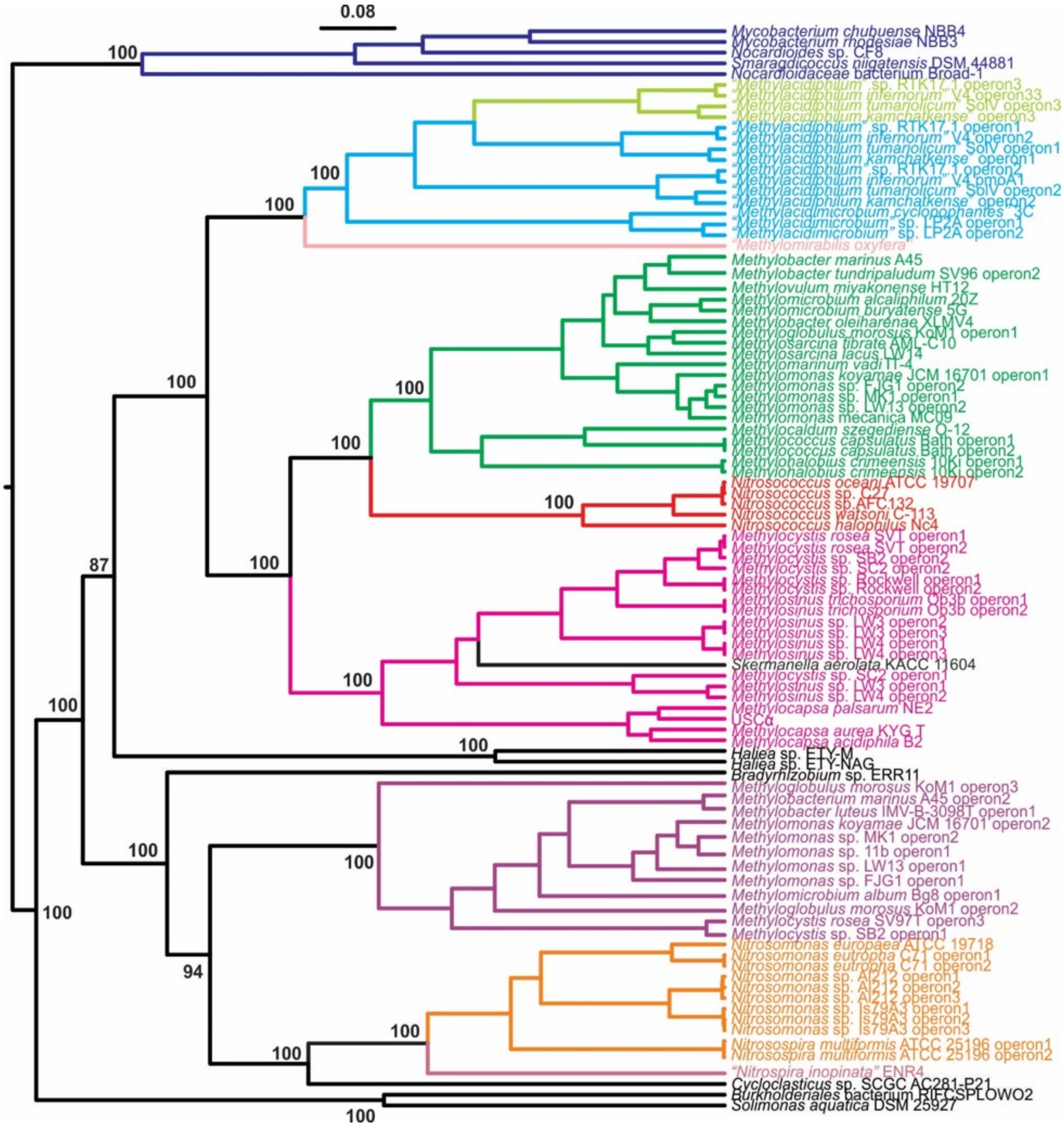

Supplementary Figure S4B. Phylogenetic tree of inferred XmoC sequences, constructed using Maximum-likelihood The tree was constructed using Seaview 4.4.12 (Gouy et al., 2010).employing LG model (Le and Gascuel, 2008) (100 iterations). Node values are based on 100 bootstrap replicates. The scale bar represents 0.2 changes per amino acid position. Colours indicate coherent functional and taxonomic groups.

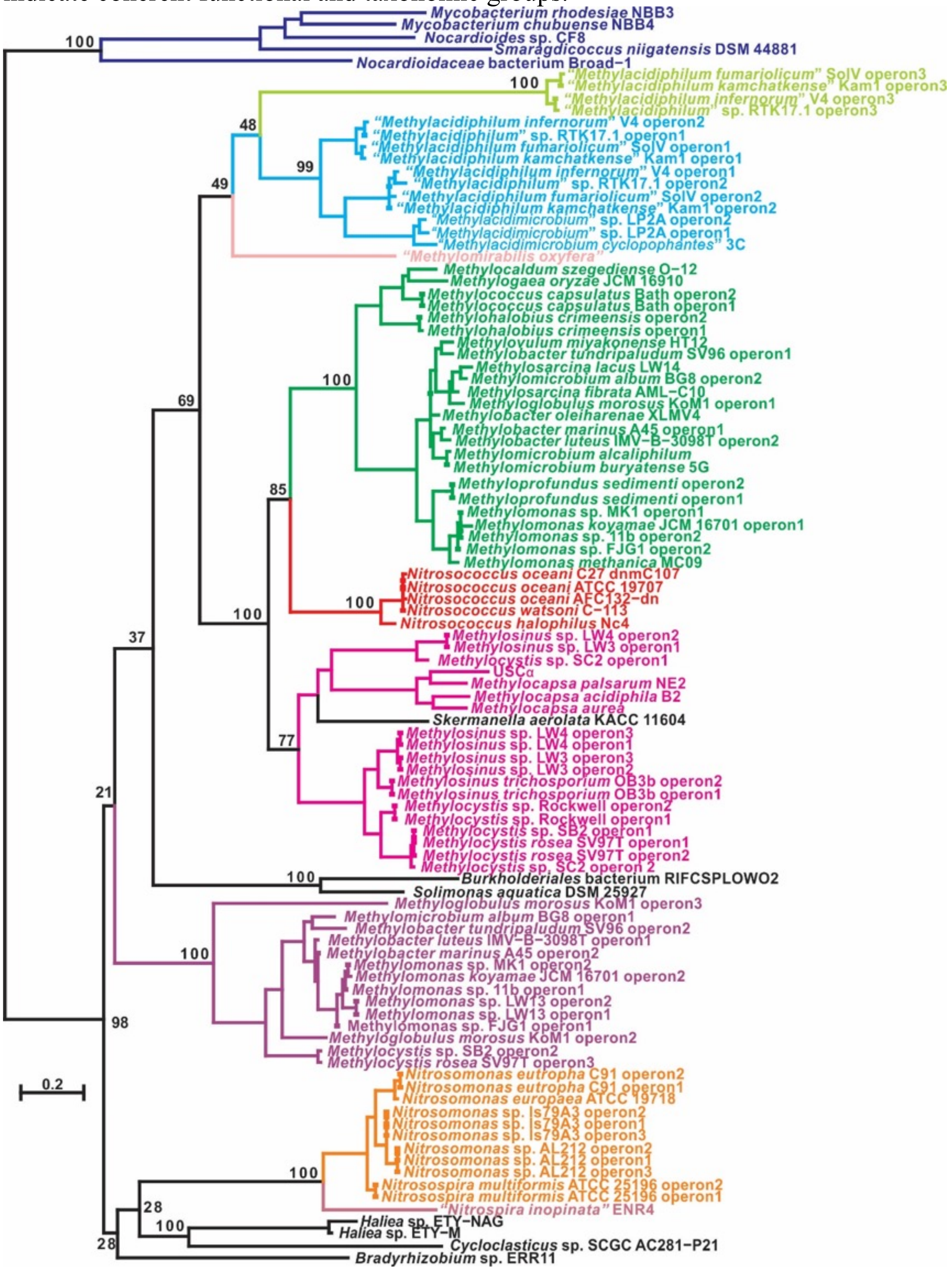

Supplementary Figure S4C. Phylogenetic tree of inferred XmoC sequences constructed using Neighbor-joining with a Poisson model in Seaview 4.4.12 (Gouy et al., 2010). Node values are based on 100 bootstrap replicates. The scale bar represents 0.2 changes per amino acid position. Colours indicate coherent functional and taxonomic groups.

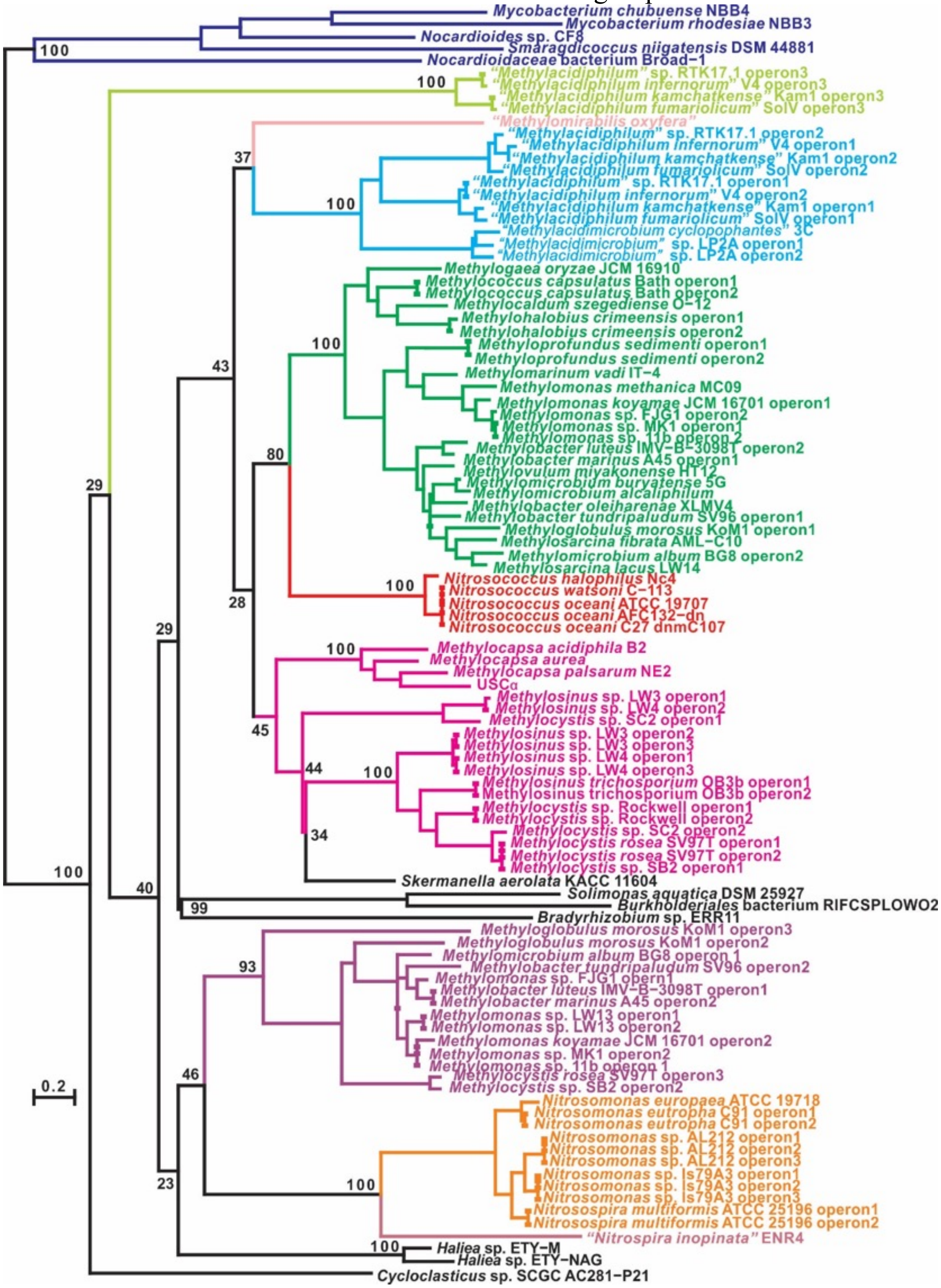

Supplementary Figure S5A. Phylogenetic tree based on inferred XmoA sequences. The tree was constructed using Bayesian analysis employing a gamma site heterogeneity model with 4 gamma categories with a relaxed clock log normal model. Node value are evolutionary rate for selected lineages (indicated as either light green or blue) based on 10,000,000 iterations, minus a burn-in of 20% of total. The light green colour indicate *pmoA* in operon3 for the *Verrucomicrobia* genus *Methylacidiphilum*.

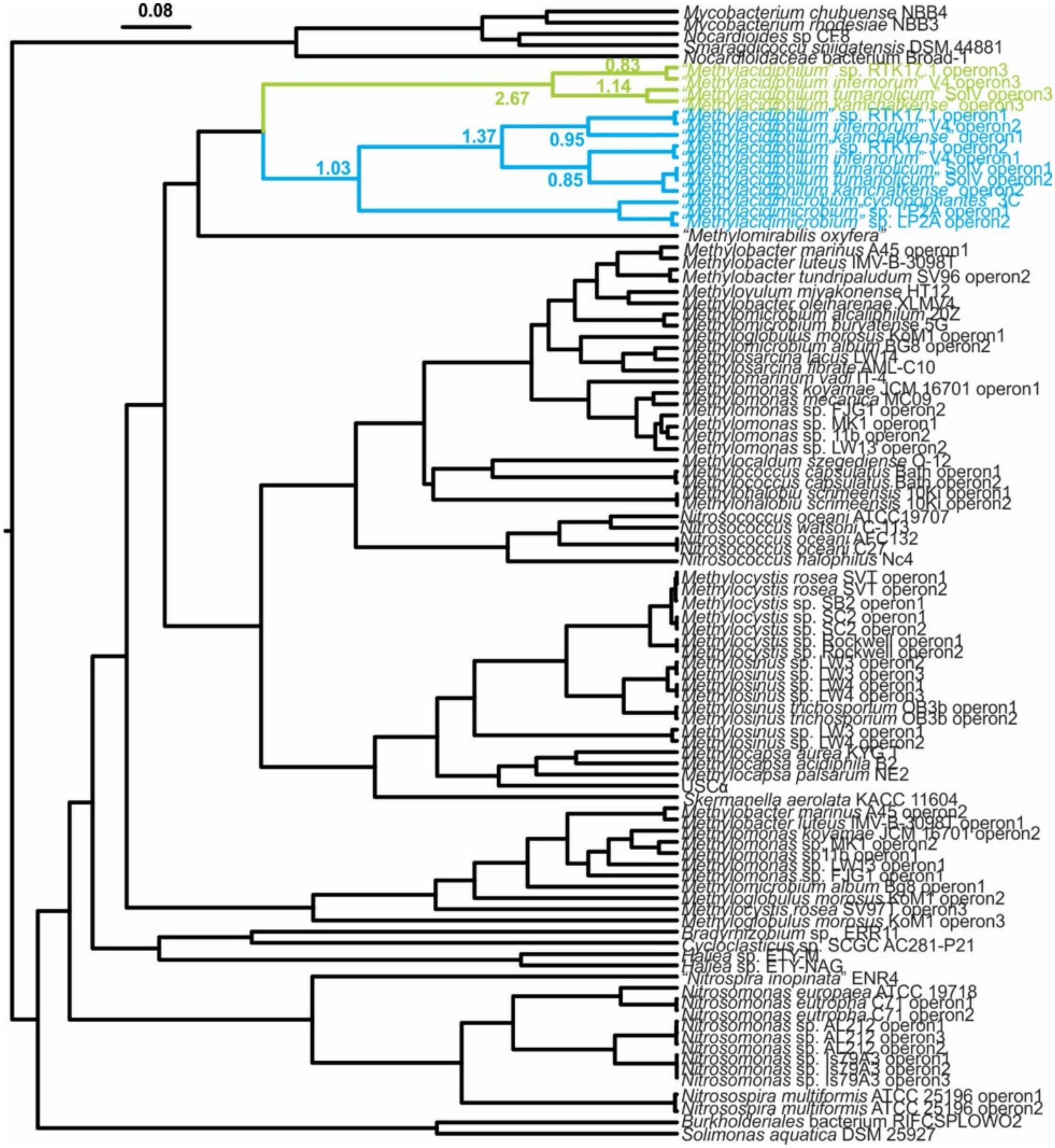

Supplementary Figure S5B. Phylogenetic tree based on inferred XmoB sequences. The tree was constructed using Bayesian analysis employing a Gamma site heterogeneity model with 4 gamma categories with a relaxed clock log normal model. Node value are evolutionary rate for lineage (indicated as either light green or blue) based on 10,000,000 iterations, minus a burn-in of 20% of total. The light green colour indicate *pmoB* in operon3 for the *Verrucomicrobia* genus *Methylacidiphilum*.

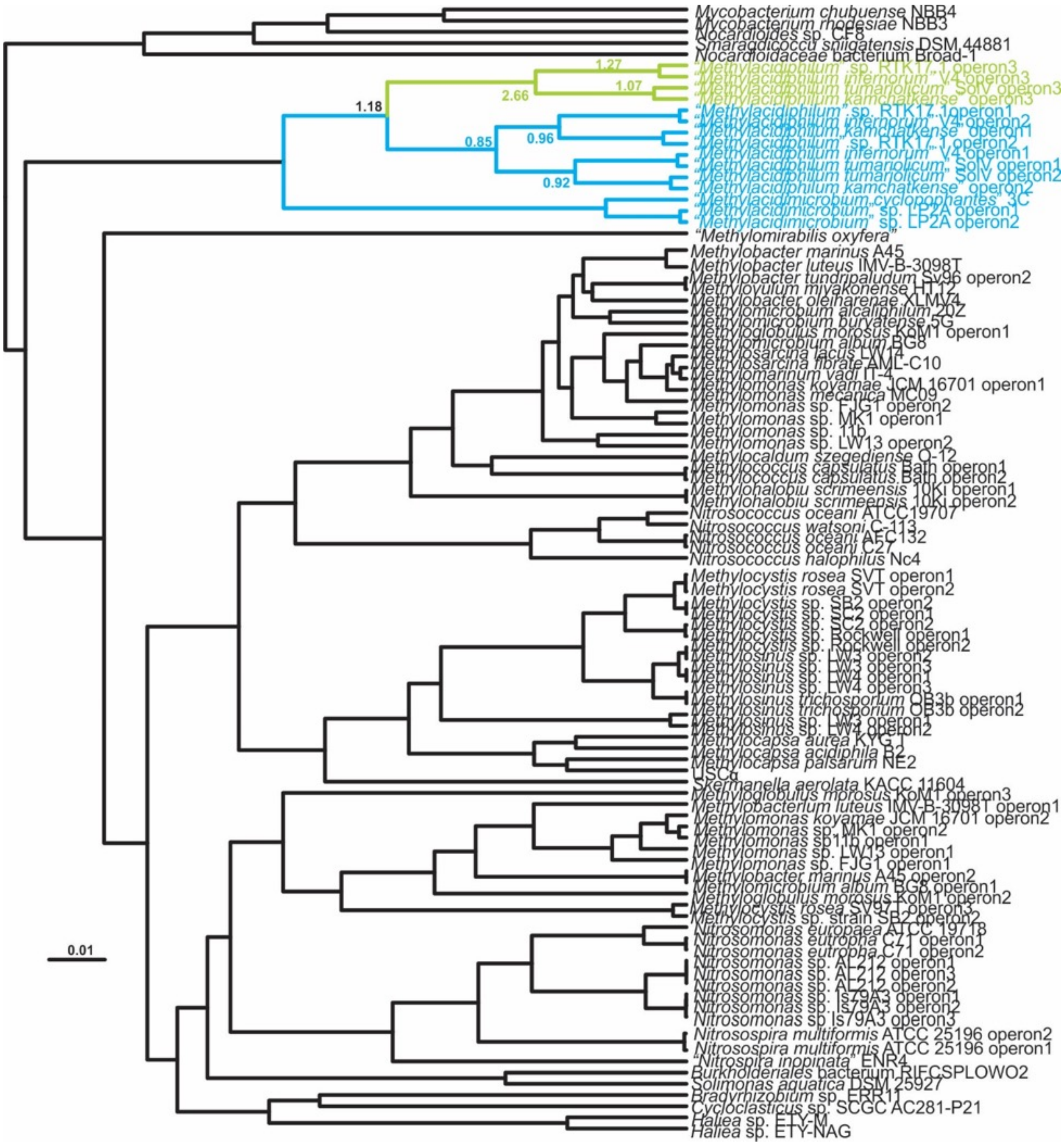

Supplementary Figure S5C. Phylogenetic tree based on inferred XmoC sequences. The tree was constructed using Bayesian analysis employing a Gamma site heterogeneity model with 4 gamma categories with a relaxed clock log normal model. Node value are evolutionary rate for lineage (indicated as either light green or blue) based on 10,000,000 iterations, minus a burn-in of 20% of total. The light green colour indicate *pmoC* in operon3 for the *Verrucomicrobia* genus *Methylacidiphilum*.

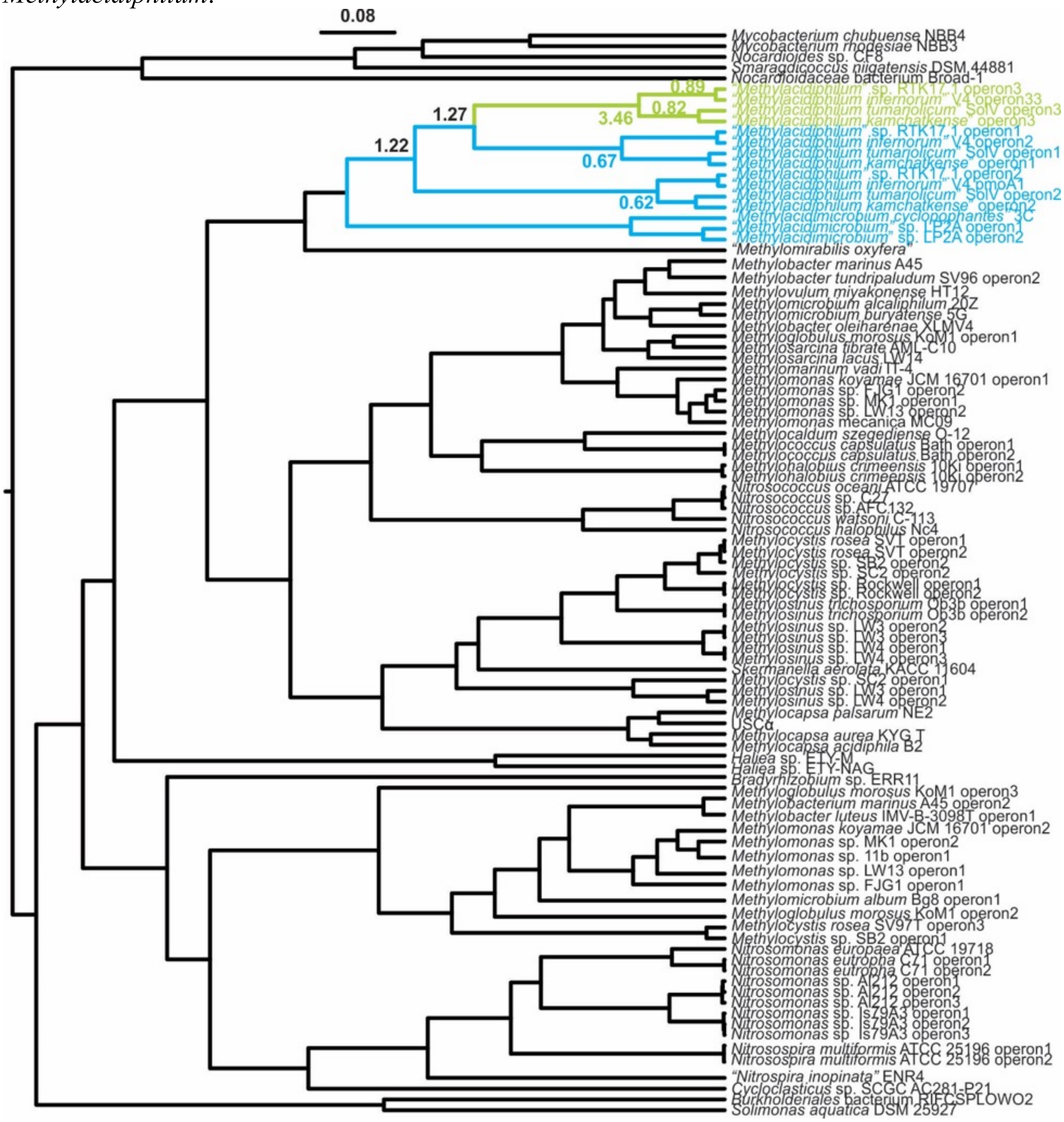

Supplementary Tables

Supplementary Table 1. Estimation of potential LGT in genomes having *xmoCAB* operons. Columns 2-4 shows G+C compositional bias in the genomes compared to the operons. Column 5 -7 shows KL divergence values between 0 to 1. Higher KL values indicate higher compositional bias of the operon compared to the host genome, and a high probability of LGT. Values close to 0 indicate low compositional bias, and a lower chance of LGT. TETRA gives an output of tetra nucleotide frequency or tetranucleotide usage patterns in a DNA sequences (Teeling et al., 2004), CodonW calculates codon usage frequency in a DNA sequences (Angellotti et al., 2007), and Alien Hunter gives an output of 2500 base pair windows based on the frequency of every k-mer from 2-8 nucleotides (Vernikos and Parkhill, 2006). BT indicates the KL calculated by Alien Hunter was below a significance threshold (i.e. not significantly greater than 0). Column 8 indicates whether the *xmoCAB* operon was estimated to be located within a genomic island using IslandViewer (Vernikos and Parkhill, 2006), placement on an island is indicated by "LGT".

Organism	Genome GC (%)	*pmoCAB* GC (%)	GC (%) Deviation	Kullback Leibler (KL) divergence			Island Viewer
				Alien Hunter	CodonW	TETRA	
***Betaproteobacteria*-nitrifiers**							
Nitrosomonas europaea ATCC 19718	50.7	48	-2.7	0.430	0.135	0.072	-
Nitrosomonas eutropha C91 operon1	48.5	47	-1.5	0.281	0.146	0.073	-
Nitrosomonas eutropha C91 operon2	48.5	46.9	-1.6	0.341	0.149	0.074	-
Nitrosomonas sp. AL212 operon1	48.8	47.6	2.8	0.246	0.212	0.071	-
Nitrosomonas sp. AL212 operon2	48.8	47.7	2.9	0.250	0.214	0.071	-
Nitrosomonas sp. AL212 operon3	48.8	47.7	2.9	0.269	0.214	0.071	LGT
Nitrosomonas sp. Is79A3 operon1	45.5	47.4	2.0	0.279	0.202	0.080	-
Nitrosomonas sp. Is79A3 operon2	45.5	47.3	1.9	0.181	0.198	0.080	-
Nitrosomonas sp. Is79A3 operon3	45.5	47.4	2.0	0.209	0.201	0.080	LGT
Nitrosospira multiformis ATCC 25196 operon1	53.9	58.8	4.9	0.169	0.211	0.084	-
Nitrosospira multiformis ATCC 25196 operon2	53.9	58.8	4.9	0.235	0.211	0.085	-
Nitrosospira multiformis ATCC 25196 operon3	53.9	58.8	4.9	0.285	0.212	0.084	-
Mean			**2.0**	**0.265**	**0.192**	**0.077**	
Gammaproteobacteria-pxm							
Methylobacter luteus IMV-B-3098 operon1	51.1	43.5	-7.6	BT	0.087	0.035	-
Methylobacter marinus A45 operon2	52.6	51.9	-0.7	BT	0.084	0.038	-
Methylomonas sp. 11B operon1	51.4	45.3	-6.1	BT	0.070	0.032	-
Methylomicrobium album BG8 operon1	56.3	48.3	-8.0	BT	0.050	0.035	-
Mean			**-5.6**	**BT**	**0.073**	**0.035**	-

Alphaproteobacteria-pxm							
Methylocystis rosea SV97 operon3	62.5	56.9	-5.6	0.184	0.077	0.072	-
Verrucomicrobia							
"Methyacidiphilum infernorum" V4 operon1	45.5	48.9	3.4	BT	0.078	0.064	LGT
"Methyacidiphilum infernorum" V4 operon2	45.5	48.1	2.6	BT	0.081	0.043	LGT
"Methyacidiphilum infernorum" V4 operon3	45.5	37.9	-7.6	0.467	0.228	0.130	-
"Methylacidimicrobium cyclopophantes" 3C	60.6	59.8	-0.7	0.206	0.111	0.059	-
"Methylacidimicrobium" sp. LP2A operon1	62.7	60.1	-2.6	0.274	0.073	0.063	-
"Methylacidimicrobium" sp. LP2A operon2	62.7	60.3	-2.4	0.274	0.073	0.059	-
Mean			**-1.2**		**0.107**	**0.070**	
**NC10**							
"*Methylomirabilis oxyfera"*	58.6	56.3	-2.3	BT	0.067	0.046	-
Beijerinckaceae							
Methylocapsa acidiphila B2	61.9	60	-1.9	0.273	0.131	0.064	-
Methylocystaceae							
Methylosinus trichosporium OB3b operon1	66	63	-1.7	0.191	0.152	0.085	-
Methylosinus trichosporium OB3b operon2	66	63	-1.7	0.163	0.151	0.084	-
Methylocystis sp. Rockwell operon1	62.8	61.3	-1.5	0.192	0.202	0.076	-
Methylocystis sp. Rockwell operon2	62.8	61.3	-1.5	0.214	0.133	0.075	-
Methylocystis sp. SC2 operon1	63.4	61.3	-2.1	0.269	0.101	0.052	-
Methylocystis sp. SC2 operon2	63.4	61.7	-1.7	0.189	0.226	0.065	-
Methylosinus sp. LW3 operon1	64.7	62.4	-2.3	0.187	0.065	0.050	-
Methylosinus sp. LW3 operon2	64.7	63	-1.7	0.148	0.184	0.109	-
Methylosinus sp. LW3 operon3	64.7	63	-1.7	0.151	0.184	0.109	-
Methylosinus sp. LW4 operon1	64.9	63.1	-1.8	0.199	0.174	0.113	-
Methylosinus sp. LW4 operon2	64.9	62.5	-2.4	0.228	0.067	0.052	-
Methylosinus sp. LW4 operon3	64.9	63.1	-1.8	0.218	0.174	0.112	-
Methylocystis rosea SV97 operon1	62.5	61.2	-1.3	BT	0.219	0.067	-
Methylocystis rosea SV97 operon2	62.5	61.2	-1.3	BT	0.219	0.068	-
Mean			**-1.8**		**0.161**	**0.080**	
Gammaproteobacteria-nitrifiers							
Nitrosococcus oceani C-107	50.3	50.4	0.1	0.198	0.088	0.035	-
Nitrosococcus watsoni C-113	50.1	50.5	0.4	0.196	0.094	0.034	-
Nitrosococcus halophilus Nc4	51.6	52.4	0.8	BT	0.072	0.055	-
Nitrosococcus AFC27	51	50.1	-0.9	BT	0.087	0.048	-
Nitrosococcus AFC132	51	49.8	-1.2	BT	0.085	0.042	-
Nitrosococcus C27	51	49.4	-1.6	BT	0.085	0.042	-
Nitrosococcus wardiae D1FHS	50.7	52.0	1.3	BT	0.243	0.135	-
Mean			**0.15**		**0.107**	**0.055**	
Gammaproteobacteria							

Methylococcus capsulatus Bath operon1	63.6	60.7	-2.9	0.194	0.190	0.065	-
Methylococcus capsulatus Bath operon2	63.6	60.8	-2.8	0.157	0.196	0.065	-
Methylocaldum szegediense O12	57.2	56	-1.2	0.217	0.307	0.072	-
Methylococcus capsulatus ATCC 19069	63.51	58.9	-4.6	BT	0.323	0.051	-
Methylohalobius crimeensis operon1	58.28	58.1	-0.1	0.145	0.215	0.094	-
Methylohalobius crimeensis operon2	58.28	58.1	-0.2	BT	0.215	0.094	-
Methylomonas methanica MC09	51.3	44.9	-6.4	0.364	0.562	0.182	-
Methylomonas sp. 11B operon2	51.4	45.3	-6.1	0.292	0.525	0.138	LGT
Methylosarcina fibrata AML-C10 operon1	54.1	46.9	-7.2	0.318	0.486	0.172	-
Methylomicrobium album BG8 operon2	56.3	48.3	-8.0	0.273	0.583	0.203	-
Methylosarcina lacus LW14 operon1	54.7	46.8	-7.9	0.283	0.244	0.201	-
Methylomicrobium buryatense 5G	48.7	44.4	-4.7	0.462	0.405	0.145	-
Methylomicrobium alcaliphilum	48.7	44.1	-4.6	0.414	0.474	0.162	-
Methylovulum miyakonense HT12 operon1	50.7	42.1	-8.6	0.296	0.287	0.190	-
Methylobacter luteus IMV-B-3098 operon2	51.1	43.5	-7.7	0.324	0.565	0.175	LGT
Methylobacter marinus A45 operon1	52.6	43.3	-9.3	0.346	0.638	0.198	-
Mean			**-5.1**		**0.388**	**0.137**	
Thaumarcheota							
Cenarchaeum symbiosum A	57.4	52.3	-5.1	BT	0.135	0.108	-
“Nitrosoarchaeum limnia” SFB1	32.5	38.4	5.9	BT	0.154	0.092	-
“Nitrosoarchaeum koreensis” MY1	32.7	38.6	6.0	0.207	0.173	0.094	-
“Nitrosopumilus” sp. BD31	32.1	33.5	1.4	BT	0.164	0.040	-
Mean			**2.3**		**0.157**	**0.088**	
***Actinobacteria* butane oxidiser**							
Nocardioides sp. CF8	70.03	63.93	-6.1	0.297	0.104	0.093	LGT
Mycobacterium chubuense NBB4	68.29	60.90	-7.3	0.290	0.172	0.104	-
Mycobacterium rhodesiae NBB3	65.49	64.41	-1.0	0.163	0.057	0.045	LGT
Smaragdicoccus niigatensis DSM 44881	64.34	62.93	-1.4	0.214	0.066	0.043	-
Nocardioidaceae bacterium Broad-1	69.55	65.66	-3.9	0.330	0.061	0.056	-
Mean			**-3.9**	**0.259**	**0.092**	**0.068**	

Supplementary Table 2A. Commonly used primers for detection of methanotrophs and their specificity to respective methane oxidisers and nitrifiers. Numbers represent the total mismatches of the primer sequences to the target DNA. A pound symbol indicates >5 mismatches.

**Organism**	***pmoA* primers***			
	**A189F**	**A682r**	**Mb661**	**650r**
***Betaproteobacteria*-nitrifiers**				
Nitrosomonas europaea ATCC 19718	1	2	#	#
Nitrosomonas eutropha C91	1	4	#	#
Nitrosomonas sp. AL212	1	3	#	#
Nitrosomonas sp. Is79A3	2	#	#	#
Nitrosospira multiformis ATCC 25196	0	2	#	#
***Gammaproteobacteria*-nitrifiers**				
Nitrosococcus oceani C-107	1	5	3	2
Nitrosococcus watsoni C-113	1	#	3	3
Nitrosococcus halophilus Nc4	1	3	3	4
Nitrosococcus AFC27	1	5	3	2
Nitrosococcus AFC132	3	#	#	4
Nitrosococcus C27	3	#	#	4
***Gammaproteobacteria*-pxm**				
Methylobacter luteus IMV-B-3098	#	#	#	#
Methylobacter marinus A45	#	#	#	#
Methylomonas sp. 11B	#	#	#	#
Methylomicrobium album BG8	#	#	#	#
***Alphaproteobacteria*-pxm**				
Methylocystis rosea SV97	#	#	#	#
Beijerinckaceae				
Methylocapsa acidiphila B2	1	2	4	#
Methylocystaceae				
Methylosinus trichosporium OB3b	0	2	2	0
Methylocystis sp. SC2	0	2	1	0

Methylosinus sp. LW3	0	2	3	0
Methylosinus sp. LW4	0	2	3	0
Methylocystis rosea SV97	0	2	1	0
Gammaproteobacteria				
Methylococcus capsulatus Bath	0	2	1	0
Methylocaldum szegediense O12	0	1	1	1
Methylohalobius crimeensis	0	2	1	1
Methylococcus capsulatus Texas	0	2	1	0
Methylomonas methanica MC09	0	3	1	1
Methylomonas sp. 11B 2	1	3	1	2
Methylosarcina fibrata AML-C10	1	3	1	2
Methylomicrobium album BG8	0	4	1	1
Methylosarcina lacus LW14	0	3	1	1
Methylomicrobium buryatense 5G	1	3	1	3
Methylomicrobium alcaliphilum	1	3	1	3
Methylovulum miyakonense HT12	1	3	1	2
Methylobacter luteus IMV-B-3098	1	3	1	2
Methylobacter marinus A45	1	3	1	2
Verrucomicrobia				
"Methyacidiphilum infernorum" V4	5	4	#	#
"Methylacidimicrobium cyclopophantes" 3C	3	1	#	#
"Methylacidimicrobium" sp. LP2A	3	1	#	#
"Methylacidiphilum fumarolicum" SolV	2	5	#	#
**NC10**				
"Methylomirabilis oxyfera"	0	#	#	#

* Primer (5'- 3') (reference) #: 5 or more mismatches

A189f: GGNGACTGGGACTTCTGG (Holmes et al., 1995)

A682r: GAASGCNGAGAAGAASGC (Holmes et al., 1995)

Mb661: CCGGMGCAACGTCYTTACC (Costello and Lidstrom, 1999)

A650r: ACGTCCTTACCGAAGGT (Bourne et al., 2001)

Supplementary Table 2B. Specificity of group-specific primers or primer pairs used to target various *xmoA* genes (especially *pmoA* and *amoA*) in previous studies. Numbers indicate the maximum number of mismatches to the target DNA of any member of a specific taxon/group. A pound symbol represents >5 mismatches.

	Group specific primers																
	**amoA-1/amaA-2IR**	**ComaA-244F/ComaA-659R**	**F326**	**r643**	**Forest675R**	**Usca-346f**	**ComaB-244F/ComaB-659R**	**II223F**	**Mcap630**	**II646R**	**Mc468R**	**Mb601R**	**V170f/V613B**	**cmo682/cmo568**	**cmo568**	**cmo182**	**HD616**
**Betaproteobacteria-nitrifiers**																	
Nitrosomonas europaea ATCC 19718	0/1	3/#	#	#	#	#	4/#	#	#	#	#	#	2/#	#	#	#	1
Nitrosomonas eutropha C91	1/0	3/#	#	#	#	#	4/#	#	#	#	#	#	2/#	#	#	#	1
Nitrosomonas sp. AL212	2/2	4/4	#	#	#	#	#	#	#	#	#	#	3/#	#	#	#	0
Nitrosomonas sp. Is79A3	1/2	4/3	#	#	#	#	#	#	#	#	#	#	3/#	#	#	#	1
Nitrosospira multiformis ATCC 25196	0/0	3/4	#	#	#	#	#	#	#	#	#	#	4/#	#	#	#	1

***Gammaproteobacteria* -nitrifiers**																	
Nitrosococcus oceani C-107	#	#	#	2	#	#	#	#	#	#	#	#	3/#	#	#	#	0
Nitrosococcus watsoni C-113	#	#	#	3	#	#	#	#	#	#	#	#	2/#	#	#	#	0
Nitrosococcus halophilus Nc4	#	#	#	3	#	#	#	4	3	#	#	#	3/#	3	#	#	0
Nitrosococcus AFC27	#	#	#	2	#	#	#	#	#	#	#	#	3/#	#	#	#	0
Nitrosococcus AFC132	#	#	#	4	#	#	#	#	#	#	#	#	2/#	#	#	#	0
Nitrosococcus C27	#	#	#	4	#	#	#	#	#	#	#	#	2/#	#	#	#	0
***Gammaproteobacteria* -pxm**																	
Methylobacter luteus IMV-B-3098	#	#	#	#	#	#	#	#	3	3	#	#	2/#	#	#	#	1
Methylobacter marinus A45	#	#	#	#	#	#	#	#	2	3	#	#	2/#	#	#	#	1
Methylomonas sp. 11B	#	#	#	#	#	#	#	#	4	4	#	#	4/#	#	#	#	1
Methylomicrobium album BG8	#	#	#	#	#	#	#	#	4	2	#	#	#	#	#	#	1
***Alphaproteobacteria*-pxm**																	
Methylocystis rosea SV97	#	#	#	#	#	#	#	#	2	4	#	#	#	#	#	#	1
Beijerinckaceae																	

Methylocapsa acidiphila B2	#	#	#	3	3	2	#	4	0	1	#	#	2/#	#	#	#	1
Methylocystaceae																	
Methylosinus trichosporium OB3b	#	#	1	2	#	#	#	0	2	0	#	#	4/#	#	#	#	0
Methylocystis sp. SC2	#	#	0	3	#	#	#	0	2	0	#	#	3/#	#	#	#	0
Methylosinus sp. LW3	#	#	0	2	#	#	#	0	2	0	4	#	#	#	#	#	0
Methylosinus sp. LW4	#	#	0	2	#	#	#	0	3	0	#	#	4/#	#	#	#	0
Methylocystis rosea SV97	#	#	0	3	#	#	#	0	2	0	#	#	3/#	#	#	#	0
Gammaproteobacteria																	
Methylococcus capsulatus Bath	#	#	0	1	#	2	#	#	#	#	0	3	#	#	#	3	0
Methylocaldum szegediense O12	#	#	#	2	#	3	#	3	#	#	1	2	#	#	#	4	0
Methylohalobius crimeensis	#	#	#	2	#	2	#	3	#	#	2	3	#	4	#	#	0
Methylococcus capsulatus Texas	#	#	0	1	#	2	#	#	#	#	0	3	#	#	#	3	0
Methylomonas methanica MC09	#	#	#	3	#	3	#	#	#	#	#	1	3/#	#	#	#	0
Methylomonas sp. 11B	#	#	#	3	#	4	#	#	#	#	#	0	3/#	#	#	#	0
Methylosarcina fibrata AML-C10	#	#	#	3	#	4	#	4	#	#	#	0	3/#	#	#	#	0

Methylomicrobium album BG8	#	#	#	2	#	3	#	4	#	#	#	0	3/#	#	#	#	0
Methylosarcina lacus LW14	#	#	#	3	#	3	#	#	#	#	#	0	3/#	#	#	#	1
Methylomicrobium buryatense 5G	#	#	#	3	#	3	#	#	#	#	#	2	2/#	#	#	#	0
Methylomicrobium alcaliphilum	#	#	#	3	#	3	#	#	#	#	#	2	2/#	#	#	#	0
Methylovulum miyakonense HT12	#	#	#	3	#	3	#	#	#	#	#	0	2/#	#	#	#	1
Methylobacter luteus IMV-B-3098	#	#	#	3	#	3	#	#	#	#	#	0	2/#	#	#	#	1
Methylobacter marinus A45	#	#	#	3	#	3	#	#	#	#	#	0	2/#	#	#	#	1
Verrucomicrobia																	
"Methyacidiphilum infernorum" V4	#	#	#	#	#	#	#	#	#	#	#	#	0/0	#	#	#	0
"Methylacidimicrobium cyclopophantes" 3C	#	#	#	#	#	#	#	#	3	#	#	#	2/2	#	#	#	1
"Methylacidimicrobium" sp. LP2A	#	#	#	#	#	#	#	#	2	#	#	#	3/2	#	#	#	1
"Methylacidiphilum fumarolicum" SolV	#	#	#	#	#	#	#	#	#	#	#	#	1/0	#	#	#	1
**NC10**																	
"Methylomirabilis oxyfera"	#	#	#	#	#	#	#	#	4	#	3	#	4/#	0/0	0	0	2

Primer (5'- 3') (reference)		
AmoA-1: GGGGTTTCTACTGGTGGT (Rotthauwe et al., 1997)	F326: TGGGGYTGGACCTAYTTCC (Fjellbirkeland et al., 2001)	V170f: GGATWGATTGGAAAGATMG (Sharp et al., 2012)
AmoA-2IR: CCCCTCIGIAAAGCCTTCTTC (Avrahami et al., 2003)	r643: CCGGCRCRACGTCCTTACC (Fjellbirkeland et al., 2001)	V613B: GCAAARCTYCTCATYGTWCC (Sharp et al., 2012)
ComaA-244F: TAYAAYTGGGTSAAYTA (Pjevac et al., 2017)	II223F: CGTCGTATGTGGCCGAC (Kolb et al., 2003)	cmo682: TCGTTCTTYGCCGGRTTT (Luesken et al., 2011)
ComaA-659R: ARATCATSGTGCTRTG (Pjevac et al., 2017)	II646R: CGTGCCGCGCTCGACCATGYG (Kolb et al., 2003)	cmo568: GATGGGGATGGAGTATGTGC (Luesken et al., 2011)
ComaB-244F: TAYTTCTGGACRTTYTA (Pjevac et al., 2017)	Mcap630: CTCGACGATGCGGAGATATT (Kolb et al., 2003)	cmo182: TCACGTTGACGCCGATCC (Luesken et al., 2011)
ComaB-659R: ARATCCARACDGTGTG (Pjevac et al., 2017)	Mc468R: GCSGTGAACAGGTAGCTGCC (Kolb et al., 2003)	Usca-346f: TGGGYGATCCTNGCNC (Degelmann et al., 2009)
Forest675R: CCYACSACATCCTTACCGAA (Kolb et al., 2003)	Mb601R: ACRTAGTGGTAACCTTGYAA (Kolb et al., 2003)	HD616: AYCWKVCKNAYRTAYTCVGG (Wang et al., 2017)

Supplementary Table 3. *xmoC*, *xmoB* and *xmo*A locus tags of sequences used in this study from NCBI/JGI database. The respective protein or concatenate xmoCAB was used for phylogenetic analysis of CuMMOs gene family.

Genome	Genome Accession no.	Locus tag		
		**PmoC**	**PmoA**	**PmoB**
"Methylacidiphilum" sp. RTK17.1 operon1	LN998017	NC58166.1	ANC58167.1	ANC58168.1
"Methylacidiphilum" sp. RTK17.1 operon2		ANC58166.1	ANC58170.1	ANC58171.1
"Methylacidiphilum" sp. RTK17.1 operon3		ANC58172.1	ANC58118.1	ANC58119.1
"Methylacidiphilum infernorum" V4 operon1	NC_010794	Minf_1508	Minf_1507	Minf_1506
"Methylacidiphilum infernorum" V4 operon2		Minf_1511	Minf_1510	Minf_1509
"Methylacidiphilum infernorum" V4 operon3		Minf_1591	Minf_1590	Minf_1589
"Methylacidiphilum fumariolicum" SolV operon1	NZ_LM997411	ABU88150	ABU88148	ABU88149
"Methylacidiphilum fumariolicum" SolV operon2		ABU88151	ABU88152	ABU88153
"Methylacidiphilum fumariolicum" SolV operon3		ABU88154	ABU88155	ABU88156
"Methylacidiphilum kamchatkense" Kam1 operon1	NZ_JQNX00000000	AFC75740	AFC75741	AFC75742
"Methylacidiphilum kamchatkense" Kam1 operon2		AFC75743	AFC75744	AFC75745
"Methylacidiphilum kamchatkense" Kam1 operon3		AFC75746	ACK55193	AFC75748
"Methylacidimicrobium cyclopophantes" 3C	NZ_KB901882	A37ADRAFT_0437	A37ADRAFT_0436	A37ADRAFT_0435
"Methylacidimicrobium" sp. LP2A operon1	NZ_JAFS01000000	G346DRAFT_1245	G346DRAFT_1244	G346DRAFT_1243
"Methylacidimicrobium" sp. LP2A operon2		G346DRAFT_1248	G346DRAFT_1247	G346DRAFT_1246
Bradyrhizobium sp. ERR11	NZ_FMAI01000000	Ga0061098_101192	Ga0061098_101193	Ga0061098_101194
Solimonas aquatica	NZ_FOFS00000000	Ga0070002_104143	Ga0070002_104144	Ga0070002_104145
Cycloclasticus sp. SCGC AC281-P21	Gs0032475	Ga0055579_00185	Ga0055579_00184	Ga0055579_00183

Burkholderiales bacterium	MERU00000000	Ga0156155_1046	Ga0156155_1046	Ga0156155_1046
Skermanella aerolata KACC 11604	NZ_AVFK01000000	Ga0069205_1047	Ga0069205_1047	Ga0069205_1047
Methylocapsa aurea	KN050804	WP_084572853.1	WP_084572913.1	WP_051953405.1
Methylocapsa palsarum NE2	NZ_FOSN01000000	Ga0116912_107	Ga0116912_107	Ga0116912_107
Methylocapsa acidiphila B2	NZ_ATYA00000000	MetacDRAFT_3746	MetacDRAFT_3747	MetacDRAFT_3748
USCα	-	CAJ01564.1	CAJ01563.1	CAJ01562.1
Methylomonas sp. FJG1 operon1	NZ_CP014476	JTDD01000035	JTDD01000035	JTDD01000035
Methylomonas sp. FJG1 operon2		JTDD01000139	JTDD01000139	JTDD01000139
Methylomonas sp. LW13 operon1	NZ_JNLB00000000	U737DRAFT_scaffold00008.8	U737DRAFT_scaffold00022.22	U737DRAFT_scaffold00022.22
Methylomonas sp. LW13 operon2		U737DRAFT_scaffold00008.8	U737DRAFT_scaffold00008.8	U737DRAFT_scaffold00008.8
Methylomonas sp. MK1 operon1	AQOV01000000	G006DRAFT_scaffold00001.1	G006DRAFT_scaffold00001.1	G006DRAFT_scaffold00001.1
Methylomonas sp. MK1 operon2		G006DRAFT_scaffold00001.1	G006DRAFT_scaffold00001.1	G006DRAFT_scaffold00001.1
Methylomonas methanica MC09	CP002738	AEF98752	AEF98753	AEF98754
Methylomonas sp. 11b operon1	NZ_KI911557	Meth11bDRAFT_3088	Meth11bDRAFT_3086	Meth11bDRAFT_3087
Methylomonas sp. 11b operon2		Meth11bDRAFT_3297	Meth11bDRAFT_3298	Meth11bDRAFT_3299
Methylomonas koyamae JCM 16701 operon1	NZ_BBCK00000000	Ga0128345_10381	Ga0128345_10382	Ga0128345_10383
Methylomonas koyamae JCM 16701 operon2		Ga0128345_10542	Ga0128345_10544	Ga0128345_10543
Methyloprofundus sedimenti operon1	NZ_LPUF01000000	WP_080524231.1	WP_080524391.1	WP_080524230.1
Methyloprofundus sedimenti operon2		OQK15279.1	OQK15452.1	OQK15278.1
Methylogaea oryzae JCM 16910	NZ_BBDL01000000	Ga0128369_1626	Ga0128369_1475	Ga0128369_1475
Methylobacter tundripaludum SV96 operon1	JH109152	EGW22255	EGW22253	EGW22254
Methylobacter tundripaludum SV96 operon2		EGW23567	EGW23568	EGW23569
Methylobacter luteus IMV-B-3098 operon1	KE386569	WP_027157618	WP_027157616	WP_027157617
Methylobacter luteus IMV-B-3098 operon2		WP_027159169	WP_027159170	WP_027159171
Methylobacter marinus A45 operon1	NZ_ARVS01000000	WP_020158145	WP_020158144	WP_020158143
Methylobacter marinus A45 operon2		WP_027147300	WP_020159526	WP_020159527

Methylobacter sp. BBA5.1 operon1	NZ_JQKS01000000	WP_020158145	WP_020158144	WP_020158143
Methylobacter sp. BBA5.1 operon2		WP_036253497	WP_020159526	WP_036253495
Methylococcus capsulatus Bath operon1	NC_002977	AAB49820	AAB49821	AAB49822
Methylococcus capsulatus Bath operon2		AAB51064	AAB51065	AAB51066
Methylocaldum szegediense O-12	NZ_ATXX01000000	WP_026609016	WP_026609851	WP_026609852
Methylohalobius crimeensis 10Ki operon1	NZ_ATXB01000000	WP_022947314	WP_022947315	WP_022947316
Methylohalobius crimeensis 10Ki operon2		WP_022949196	WP_022947315	WP_022947316
Methyloglobulus morosus KoM1 operon1	NZ_AYLO00000000	MKO_MKO1_c77.74	MKO_MKO1_c77.74	MKO_MKO1_c77.74
Methyloglobulus morosus KoM1 operon2		MKO_MKO1_c123.115	MKO_MKO1_c123.115	MKO_MKO1_c123.115
Methyloglobulus morosus KoM1 operon3		MKO_MKO1_c167.152	MKO_MKO1_c167.152	MKO_MKO1_c167.152
Methylomarinum vadi IT-4	NZ_JPON01000000	EP25DRAFT_1965	EP25DRAFT_1964	EP25DRAFT_1963
Methylomicrobium agile ATCC 35068 operon1	JPOJ01000000	CC94DRAFT_0956	CC94DRAFT_0955	CC94DRAFT_0954
Methylomicrobium agile ATCC 35068 operon2		CC94DRAFT_2803	CC94DRAFT_2805	CC94DRAFT_2804
Methylomicrobium album BG8 operon 1	CM001475	EIC29217	EIC29218	EIC29219
Methylomicrobium album BG8 operon 2		EIC31240	EIC31239	EIC31238
Methylomicrobium alcaliphilum	NC_016108	CCE22212	CCE22213	CCE22214
Methylomicrobium buryatense 5G		WP_017840377	WP_017840378	WP_017840379
Methylocystis rosea SV97 operon1	ARCT01000000	WP_026222791	WP_014892304	WP_018408664
Methylocystis rosea SV97 operon2		WP_026222791	WP_014892304	WP_018408664
Methylocystis rosea SV97 operon3		WP_018409559.1	WP_026223175.1	WP_018409560
Methylocystis sp. SB2 operon 1	NZ_AYNA01000000	WP_014889687	WP_014889688	WP_014889689
Methylocystis sp. SB2 operon 2		WP_014890337	WP_014892304	WP_014892305
Methylocystis sp. Rockwell ATCC 49242 operon1	NZ_KE124774	WP_084678888	WP_036281738	WP_036287217
Methylocystis sp. Rockwell ATCC 49242 operon2		WP_084678888	WP_036281738	WP_036287217
Methylocystis sp. SC2 operon1	NC_018485	CCJ05653	CCJ05654	CCJ05655
Methylocystis sp. SC2 operon2		CCJ08277	CCJ08278	CCJ08279

Methylocystis sp. SC2 operon3		CCJ08984	CCJ08985	CCJ08986
Methylosinus trichosporium OB3b operon1	NZ_ADVE02000000	ATQ66668	ATQ66669	ATQ70154
Methylosinus trichosporium OB3b operon2		ATQ68210	ATQ68209	ATQ70325
Methylosinus sp. LW4 operon1	KB900626	WP_018265151	WP_018265152	WP_043332230
Methylosinus sp. LW4 operon2		WP_018265988	WP_018265987	WP_018265986
Methylosinus sp. LW4 operon3		WP_018265151	WP_018265152	WP_043332230
Methylovulum miyakonense HT12	NZ_KB913025	BAJ17640	BAJ17641	BAJ17642
Methylosinus sp. LW3 operon1	NZ_AZUO01000000	WP_024879510	WP_024879511	WP_024879512
Methylosinus sp. LW3 operon2		WP_024880171	WP_018265152	WP_051465414
Methylosinus sp. LW3 operon3		WP_024880171	WP_018265152	WP_051465414
Nitrosococcus oceani ATCC 19707	NC_007484	WP_011330983	WP_011330982	WP_002811287
Nitrosococcus oceani C-27	NZ_JPGN01000000	WP_036526307	WP_011330982	WP_002811287
Nitrosococcus oceani AFC132	NZ_JPFN01000000	WP_036502511	WP_011330982	WP_036502509
Nitrosococcus halophilus Nc4	NC_013960	ADE13855	ADE13856	ADE13857
Nitrosococcus watsoni C-113	NC_014315	WP_013219692	WP_013219693	WP_013219694
Mycobacterium chubuense NBB4	NC_018027	AFM20520	AFM20519	AFM20518
Mycobacterium rhodesiae NBB3	NC_016604	WP_014211367.1	WP_014211362.1	WP_014211290.1
Smaragdicoccus niigatensis DSM 44881	NZ_AQXZ01000000	F600DRAFT_00579	F600DRAFT_00580	F600DRAFT_00581
Nocardioides sp. CF8	NZ_CM001852	EON22009	EON22010	EON22011
Nocardioidaceae bacterium Broad-1	GL873260	EGD43188	EGD43187	EGD43186
"*Nitrosoarchaeum limnia*" SFB1	AEGP01000000	EGG41086	EGG41084	EGG41085
"*Nitrosopumilus salaria*" BD31	NZ_AEXL02000000	EIJ67027	EIJ67028	EIJ67026
"*Nitrosopumilus koreensis*" MY1	NZ_AFPU01000000	AFS81497	AFS81495.1	AFS81498.1
Cenarchaeum symbiosum A	DP000238	ABK77035	ABK77038	ABK77030
Nitrosomonas europaea ATCC 19718	NC_004757	WP_011111554	WP_041357108	WP_011111552
Nitrosomonas eutropha C91 operon1	NC_008344	WP_011635098	WP_041353839	WP_011635096
Nitrosomonas eutropha C91 operon2		WP_011635098	WP_041353839	WP_011635096

Nitrosomonas sp. AL212 operon1	NC_015222	WP_013646791	WP_013646792	WP_013646793
Nitrosomonas sp. AL212 operon2		WP_013646791	WP_013646792	WP_013646793
Nitrosomonas sp. AL212 operon3		WP_013646791	WP_013646792	WP_013646793
Nitrosomonas sp. Is79A3 operon1	NC_015731	WP_013964653	WP_013964654	WP_013964655
Nitrosomonas sp. Is79A3 operon2		WP_013964653	WP_013964654	WP_013964655
Nitrosomonas sp. Is79A3 operon3		WP_013964653	WP_013964654	WP_013964655
Nitrosospira multiformis ATCC 25196 operon1	NC_007614	WP_011379539	WP_011380154	WP_011380155
Nitrosospira multiformis ATCC 25196 operon2		WP_011379539	WP_011380154	WP_011380155
"*Nitrospira inopinata"* ENR4	NZ_LN885086	WP_062484140	WP_062484767	WP_062484768
Haliea sp. ETY-M	-	BAM38052	BAM38053	BAM38054
Haliea sp. ETY-NAG	-	BAM38058	BAM38059	BAM38060
"*Methylomirabilis oxyfera"*	FP565575	CBE69521	CBE69519	CBE69517

Supplementary References

Angellotti, M.C., Bhuiyan, S.B., Chen, G., and Wan, X.-F. (2007). CodonO: codon usage bias analysis within and across genomes. *Nucleic Acids Research.* 35**,** W132-W136. doi:10.1093/nar/gkm392

Avrahami, S., Liesack, W., and Conrad, R. (2003). Effects of temperature and fertilizer on activity and community structure of soil ammonia oxidizers. *Environ. Microbiol.* 5**,** 691-705.

Bourne, D.G., Mcdonald, I.R., and Murrell, J.C. (2001). Comparison of *pmoA* PCR primer sets as tools for investigating methanotroph diversity in three Danish soils. *Appl. Environ. Microbiol.* 67**,** 3802-3809.

Costello, A.M., and Lidstrom, M.E. (1999). Molecular characterization of functional and phylogenetic genes from natural populations of methanotrophs in lake sediments. *Appl. Environ. Microbiol.* 65**,** 5066-5074.

Degelmann, D.M., Borken, W., and Kolb, S. (2009). Methane oxidation kinetics differ in European beech and Norway spruce soils. *Eur J Soil Sci.* 60**,** 499-506. doi:10.1111/j.1365-2389.2009.01138.x

Fjellbirkeland, A., Torsvik, V., and Ovreas, L. (2001). Methanotrophic diversity in an agricultural soil as evaluated by denaturing gradient gel electrophoresis profiles of *pmoA*, *mxaF* and 16S rDNA sequences. *Antonie Van Leeuwenhoek.* 79**,** 209-217.

Gouy, M., Guindon, S., and Gascuel, O. (2010). SeaView version 4: A multiplatform graphical user interface for sequence alignment and phylogenetic tree building. *Mol. Biol. Evol.* 27**,** 221-224. doi:10.1093/molbev/msp259

Holmes, A.J., Costello, A., Lidstrom, M.E., and Murrell, J.C. (1995). Evidence that participate methane monooxygenase and ammonia monooxygenase may be evolutionarily related. *FEMS Microbiol. Lett.* 132**,** 203-208. doi:10.1111/j.1574-6968.1995.tb07834.x

Kolb, S., Knief, C., Stubner, S., and Conrad, R. (2003). Quantitative detection of methanotrophs in soil by novel *pmoA*-targeted real-time PCR assays. *Appl. Environ. Microbiol.* 69**,** 2423-2429.

Le, S.Q., and Gascuel, O. (2008). An improved general amino acid replacement matrix. *Mol. Biol. Evol.* 25**,** 1307-1320. doi:10.1093/molbev/msn067

Luesken, F.A., Zhu, B., Van Alen, T.A., Butler, M.K., Diaz, M.R., Song, B., Op Den Camp, H.J., Jetten, M.S., and Ettwig, K.F. (2011). pmoA Primers for detection of anaerobic methanotrophs. *Appl. Environ. Microbiol.* 77**,** 3877-3880. doi:10.1128/AEM.02960-10

Pjevac, P., Schauberger, C., Poghosyan, L., Herbold, C.W., Van Kessel, M., Daebeler, A., Steinberger, M., Jetten, M.S.M., Lucker, S., Wagner, M., and Daims, H. (2017). AmoA-targeted polymerase chain reaction primers for the specific detection and quantification of comammox *Nitrospira* in the environment. *Front. Microbiol.* 8**,** 1508. doi:10.3389/fmicb.2017.01508

Rotthauwe, J.H., Witzel, K.P., and Liesack, W. (1997). The ammonia monooxygenase structural gene *amoA* as a functional marker: molecular fine-scale analysis of natural ammonia-oxidizing populations. *Appl. Environ. Microbiol.* 63**,** 4704-4712.

Sharp, C.E., Stott, M.B., and Dunfield, P.F. (2012). Detection of autotrophic verrucomicrobial methanotrophs in a geothermal environment using stable isotope probing. *Front. Microbiol.* 3**,** 303. doi:10.3389/fmicb.2012.00303

Teeling, H., Waldmann, J., Lombardot, T., Bauer, M., and Glockner, F.O. (2004). TETRA: a web-service and a stand-alone program for the analysis and comparison of tetranucleotide usage patterns in DNA sequences. *BMC Bioinformatics.* 5**,** 163. doi:10.1186/1471-2105-5-163

Vernikos, G.S., and Parkhill, J. (2006). Interpolated variable order motifs for identification of horizontally acquired DNA: revisiting the *Salmonella* pathogenicity islands. *Bioinformatics.* 22**,** 2196-2203. doi:10.1093/bioinformatics/btl369

Wang, J.G., Xia, F., Zeleke, J., Zou, B., Rhee, S.K., and Quan, Z.X. (2017). An improved protocol with a highly degenerate primer targeting copper-containing membrane-bound monooxygenase genes for community analysis of methane- and ammonia-oxidizing bacteria. *FEMS Microbiol. Ecol.* 93. doi:10.1093/femsec/fiw244
